# Environmental Temperature Controls Accumulation of Transacting siRNAs Involved in Heterochromatin Formation

**DOI:** 10.3390/genes9020117

**Published:** 2018-02-21

**Authors:** Marcello Pirritano, Ulrike Götz, Sivarajan Karunanithi, Karl Nordström, Marcel H. Schulz, Martin Simon

**Affiliations:** 1 Molecular Cell Dynamics, Centre for Human and Molecular Biology, Saarland University, 66123 Saarbrücken, Germany; s9mopirr@stud.uni-saarland.de (M.P.); U.Goetz@hohenstein.de (U.G.); 2 Cluster of Excellence, Multimodal Computing and Interaction, Saarland University and Department for Computational Biology and Applied Algorithmics, Max Planck Institute for Informatics, Saarland Informatics Campus, 66123 Saarbrücken, Germany; skarunan@mmci.uni-saarland.de (S.K.); mschulz@mmci.uni-saarland.de (M.H.S.); 3 Genetics/Epigenetics, Centre for Human and Molecular Biology, Saarland University, 66123 Saarbrücken, Germany; karl.nordstroem@uni-saarland.de

**Keywords:** RNA interference, transitivity, chromatin, environment

## Abstract

Genes or alleles can interact by small RNAs in a homology dependent manner meaning that short interfering (siRNAs) can act in *trans* at the chromatin level producing stable and heritable silencing phenotypes. Because of the puzzling data on endogenous paramutations, their impact contributing to adaptive evolution in a Lamarckian manner remains unknown. An increasing number of studies characterizes the underlying siRNA accumulation pathways using transgene experiments. Also in the ciliate *Paramecium tetraurelia*, we induce *trans* silencing on the chromatin level by injection of truncated transgenes. Here, we characterize the efficiency of this mechanism at different temperatures showing that silencing of the endogenous genes is temperature dependent. Analyzing different transgene constructs at different copy numbers, we dissected whether silencing efficiency is due to varying precursor RNAs or siRNA accumulation. Our data shows that silencing efficiency correlates with more efficient accumulation of primary siRNAs at higher temperatures rather than higher expression of precursor RNAs. Due to higher primary levels, secondary siRNAs also show temperature dependency and interestingly increase their relative proportion to primary siRNAs. Our data shows that efficient *trans* silencing on the chromatin level in *P. tetraurelia* depends on environmental parameters, thus being an important epigenetic factor limiting regulatory effects of siRNAs.

## 1. Introduction

Next to the post-transcriptional inactivation of gene expression by short interfering RNA (siRNA) or micro RNAs (miRNA), epigenetic silencing of genes occurs on the chromatin level involving siRNAs targeting loci for histone modifications in a homology dependent manner. Strikingly, this kind of co-transcriptional silencing represents a self-enforcing feedback mechanism, thus enabling self-replicating epigenetic states [1]. In addition, trans-generational epigenetic gene silencing by small RNAs has been shown in many model systems ranging from ciliates to plants and animals, although such mechanisms in mammals are still under debate [2,3]. However, heritable epigenetic silencing could be a powerful adaptation processes in a Lamarckian manner.

The ability of siRNAs to act in *trans* to another allele or homologous locus appears to be restricted. In *Schizosaccharomyces pombe*, for instance, gene position effects as well as siRNA abundance are important factors [4,5]. Therefore, many individual parameters need to be taken into account whether siRNAs can act in *trans* or not. Next to the individual characteristics of a specific pathway including its genetic requirements and the amount of precursor produced, environmental issues also need to be considered. Paramutations, meaning the heritable silencing of homologous alleles are quantitative events being sensitive to environmental stressors or growth conditions [6]. Also in animals (*Drosophila melanogaster* and *Caenorhabditis elegans*), *trans* silencing by siRNAs in a paramutative manner has been observed [7,8]. Although *trans* silencing and paramutation can be followed in *C. elegans* using different exogenic reporters, many endogenous genes are protected from silencing although a subset of genes indeed follows the behavior of the exogenic transgene reporters [7]. Although these findings tempt speculation on the evolutionary impact of this kind of quantitative epigenetics, the molecular basis of quantitative silencing is hardly understood in this context. As induced epigenetic modifications and their potential trans-generational persistence cannot be a general genome wide phenomenon rather than limited to individual plastic genes, we need to understand what makes genes sensitive for *trans* silencing, thus discriminating between epigenetic stability or variability [9].

*Paramecium tetraurelia* is a unicellular model in genetics and epigenetics [10]. RNA interference (RNAi) can be triggered by feeding of dsRNA producing bacteria similar to *C. elegans* [11] or by injection of truncated transgenes [12,13,14]. Both pathways apparently differ in their siRNAs and genetic requirements, although individual components such as Dicer1 (DCR1) and RNA dependent RNA polymerase 2 (RDR2) are shared [15,16,17]. Silencing endogenous genes by injection of transgenes producing incomplete mRNA triggers accumulation of 23 nt siRNAs. These primary (1${}^{\circ }$) siRNAs can act in *trans* to endogenous remote loci triggering loss of activating H3K4me3 and H3K9ac and accumulation of H3K27me3. Similar to the mechanism of co-transcriptional silencing, the remote locus then produces secondary (2${}^{\circ }$) siRNAs with strong antisense bias and 23 nt lenght with decreasing coverage from the 5${}^{\prime }$- to the 3${}^{\prime }$- end of the open reading frame [17]. Although we know that silencing of DCR1, RDR2, RDR3, PTIWI13 and PTIWI14 reduced the amount of 1${}^{\circ }$ and 2${}^{\circ }$ siRNAs, we still cannot dissect which components are responsible for 2${}^{\circ }$ siRNA accumulation [17]. In addition, the precise role of the two PIWI proteins in 1${}^{\circ }$ siRNA accumulation remains unclear. We show in this paper that this process depends on the environmental temperature. In contrast to homoiothermic animals, *P. tetraurelia* is a poikilothermic species. Thus, this single cell has to guarantee that all metabolic pathways run properly at the environmental temperature similar to the problems that plants and several homoiothermic animals face. It has been known for a long time that paramecia tolerate a broad spectrum of temperatures ranging from 4 ${}^{\circ }$C to almost 37 ${}^{\circ }$C for stable cultivation [18,19]. Our data here indicate that the RNAi machinery rather than the transcription of a differential precursor is responsible for differential siRNA accumulation at different environmental temperatures.

## 2. Materials and Methods

### 2.1. Cell Culture, Transgenic Lines and Phenotypic Characterization

*Paramecium tetraurelia* strain 51 was cultivated in wheat grass powder medium (WGP, Pines International Co., Lawrence, KS, USA) supplemented with 0.8 μg/mL β-sitosterol and inoculated with *Klebsiella pneumoniae*. Transformation of single cells was performed by using microinjection as described before [15]. The pTI- and pTI-/- constructs were described in [17]. Successfully transfected cells were validated by GFP expression and cultivated at 4 ${}^{\circ }$C to build up a stock and prevent autogamy. Cells of a transgenic line were split and slowly adapted to the indicated experimental temperature (18 ${}^{\circ }$C, 26 ${}^{\circ }$C, 31 ${}^{\circ }$C). The efficiency of the *ND169* gene silencing triggered by the transfected construct was observed by trichocyst discharge using picric acid.

### 2.2. DNA Isolation and Southern Blots

Intact macronuclear chromosomes were isolated by lysing cells in 0.5 M EDTA pH 9; 1% sarcosyl; 1% SDS; 0.25 mg/mL Proteinase K and incubation at 55 ${}^{\circ }$C over night. After phenol extraction and dialysis against TE buffer for 48 h, the DNA was digested with RNAse A and additionally extracted with phenol. Agarose gel electrophoresis and Southern blots were carried out by standard capillary procedures including depurination.

### 2.3. Total RNA Isolation and Northern Blots

Probes for hybridizations to long nucleic acids were generated by PCR products (nucleotide position 701-1733 for *ND169* and position 12-559 for *GFP*). Biotin labeling using Klenow exo- and random decamers was carried out using the BiotinDeca Labeling Kit (Thermo Fisher, Waltham, MA, USA). Hybridizations to Northern and Southern blots were carried out at 60 ${}^{\circ }$C in Church buffer. Signals were visualized by alkaline phosphatase (Biotin Chromogenic detection Kit, Thermo Fisher).

### 2.4. Non-Radioactive Labeling and Hybridizations

Probes for hybridizations to long nucleic acids were generated by PCR products (nucleotide position 701-1733 for *ND169* and position 12-559 for *GFP*). Biotin labeling using Klenow exo- and random decamers was carried out using the BiotinDeca Labeling Kit (Thermo Fisher, Waltham, MA, USA). Hybridizations to Northern and Southern blots were carried out at 60 ${}^{\circ }$C in Church buffer. Signals were visualized by alkaline phosphatase (Biotin Chromogenic detection Kit, Thermo Fisher).

### 2.5. Small Interfering RNA Northern Blot Analysis

For small RNA Northern blots, 50 μg total RNA were separated on a 40cm urea polyacrylamid gel (15%, 19:1 acrylamid:bis) and vacuum blotted in 20xSSC to Hybond N^+^ membranes (GE/Amersham). Radiolabeling of probes was carried out with [α-^32^P] dCTP (3000 Ci/mmol) using random octamers and Klenow exo-. Oligos complementary to the marker and Gln tRNA were 5${}^{\prime }$-labeled with T4 polynucleotid kinase using [γ-^32^P] ATP (3000 Ci/mmol) as described before [15]. Signals were detected using a Typhoon Trio Phosphoimager system (GE, Munich, Germany). Primers for the vector specific probe of the pTI- plasmid from position 817-2543 relative to the *GFP* start codon in the pTI- plasmid.

### 2.6. Short RNA Library Preparation, Illumina Sequencing and Bioinformatics Analyses

Small RNAs (17–25 nt size) from 20 μg total RNA were size selected by gel extraction using a 17.5% urea acrylamid mini-gel, visualized with SYBR-Gold (Life-Technologies, Darmstadt, Germany). Gel slices were cut and RNA was eluted by overnight incubation in 3 Vol. using 0.3M NaCl at 4 ${}^{\circ }$C, RNA was precipitated using 3 Vol. Ethanol (100%) and Glycogen (70 ng/μL) and afterwards subjected to library preparation using the NEBNext small RNA library prep Kit (New England Biolabs, Frankfurt a.M., Germany), according to the manufacturer’s instructions (3${}^{\prime }$-adapter ligation extended to 18h at 16 ${}^{\circ }$C). Sequencing was done on the Illumina HiSEQ 2500 platform (Illumina, San Diego, CA, USA) using the RAPID mode and 30 nt read length. Reads (1.9; 6.7; 5.5 million Mac mapping reads, respectively) were de-multiplexed and adapter sequences were trimmed using Trim Galore [20] that uses Cutadapt [21] with a stringency cutoff of 10. SmallRNA read alignments to transgenes and normalization of reads were carried out precisely as described in [17]. The complete analysis was done using the RAPID pipeline [22].

### 2.7. Long RNA Library Preparation and Sequencing

For long RNA Library preparation, 8 μg total RNA was treated with DNaseI (2.5U, Qiagen, Hilden, Germany), purified and precipitated using acidic phenol extraction. Furthermore, 1 μg of the DNA-free RNA was poly-A-enriched using the NEBNext Poly(A) mRNA Magenetic Isolation Module and then subjected to library preparation (NEBNext Ultra Directional RNA Library Prep Kit for Illumina) using 13 PCR cycles. Reads were quality and adapter trimmed (see above) and mapped to the *P. tetraurelia* Mac genome using STARaligner [23]. Because of the high dynamic range of transcriptomes and temperature dependent GFP over-expression, read coverages were normalized against housekeeping genes instead of TPM/RPM (Transcripts/reads per million) normalization.

## 3. Results

### 3.1. Silencing Phenotypes by Truncated Transgenes Depend on the Environmental Temperature

As transgenes injected into the somatic macronucleus are lost when cells start undergoing autogamy or conjugation, the transgene is maintained only in a single generation. Thus, after injection of young cells, transgene cultures are usually maintained in stocks at low temperatures (4–6 ${}^{\circ }$C) to limit cell divisions, which is the main factor defining clonal age for ciliates [24].

**Figure 1:**
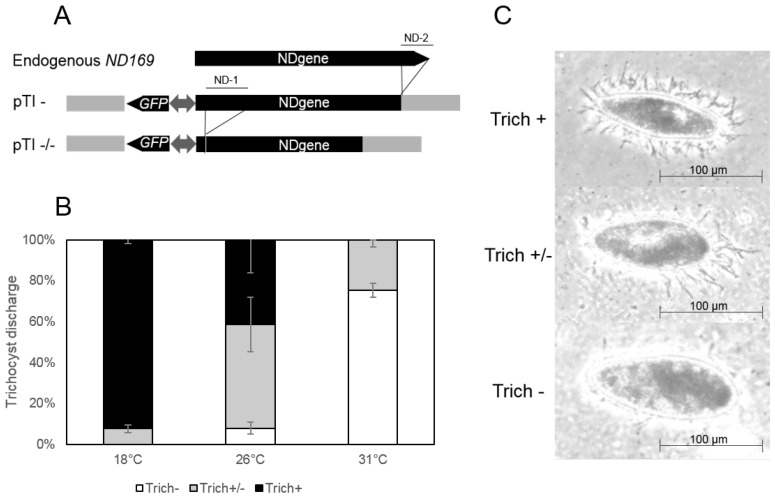
Transgene constructs and phenotypes of transgene-induced silencing. (**A**) overview of constructs. Shown are the endogenous version of the gene *ND169* (upper part), the pTI- construct, a 3${}^{\prime }$-coding region truncated version of the *ND169* gene (ND-2) and the pTI-/- construct, with additional deletions of the 5${}^{\prime }$-coding region (ND-1). Both constructs also contain a *GFP* gene under control of a bidirectional promoter; (**B**) quantification of trichocyst discharge phenotypes in pTI-/- lines at the indicated temperatures. Shown are percentages of cells with different categories of trichocyst ejection, ranging between a complete loss of gene expression (Trich-), an intermediate phenotype (Trich+/-) and no silencing (Trich+). Standard deviation and proportions of the different phenotypes are means of three biological replicates, respectively; (**C**) example of the observed trichocyst phenotypes. Phase contrast pictures show wildtype (WT) cells with full trichocyst discharge (upper panel, Trich+), a partial phenotype with few visible trichocysts (middle panel, Trich+/-) and cells showing efficient silencing and no trichocyst discharge after treatment with picric acid (lower panel, Trich-).

When going back to stock cultures of constructs that induce homology dependent gene silencing of endogenous remote loci, we realized that these do not show silencing phenotypes and are rather wild type at low temperatures. Figure 1A shows plasmid maps of two transgene constructs (pTI- and pTI-/-) containing truncated versions of the *ND169* gene. This gene was frequently used as a reporter gene, as it found to be constitutively expressed during vegetative growth. Its gene product is necessary for membrane fusion during ejections of trichocysts, thus *ND169* silenced or mutant cells cannot eject the still existing trichocysts [25].The truncated versions have been shown to accumulate long aberrant RNA triggering siRNA accumulation acting in *trans* to the endogenous *ND169* locus [15,17]. Figure 1B shows that the phenotype mediated by the transgene induced silencing of the *ND169* gene increases with the temperature in cultures maintained from 18–31 ${}^{\circ }$C indicated by a higher proportion of cells with no trichocyst discharge (Figure 1C). We consequently asked for the reason of the temperature dependency of *trans* silencing in *P. tetraurelia* and subsequently analyzed siRNA accumulation as well as precursor RNA abundance to gain insight into the molecular background.

### 3.2. Cold Temperatures Impede Short Interfering RNA Accumulation

**Figure 2:**
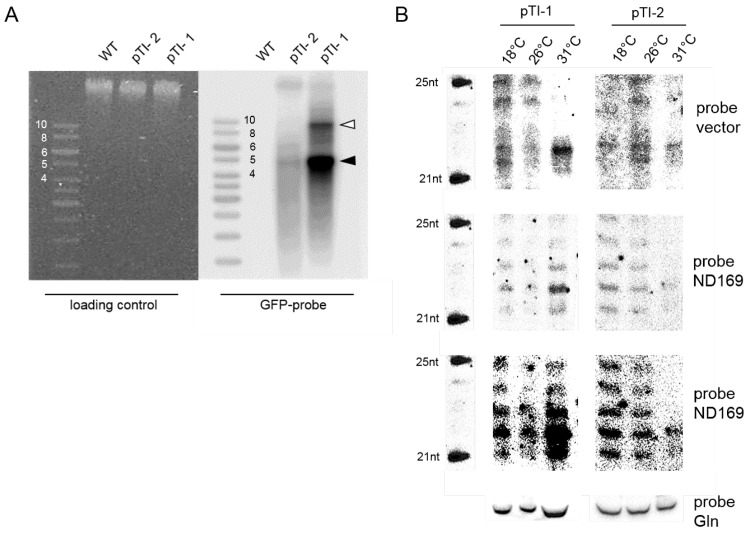
Analysis of temperature dependent siRNAs in low and high copy number injected transgenic lines. (**A**) Southern blot of macronuclear DNA isolated from WT and two pTI- lines (pTI-1, pTI-2) hybridized with a *GFP* specific probe (right). The ethidium bromide stained gel before blotting is shown on the left. The black arrowhead points to transgene mini-chromosomes in the size of the linearized plasmid of approx. 4475 bp with additional telomers. The open arrowhead points to transgene dimers resulting from endogenous end repair. Size of the ladder bands are displayed in kb; (**B**) Northern blot of siRNAs of the two transgene lines cultivated at the indicated temperatures. The marker is shown on the left (5’-OH), a probe against the glutamin tRNA serves as a loading control. The upper panel shows hybridizations with a vector specific probe, downstream of the truncated *ND169* gene region. Below, hybridizations with a *ND169* specific probe are shown with low exposure and beneath with longer exposure.

We first asked for the accumulation of siRNAs. Molecular analysis was carried out for two transgenic pTI- lines with either low or high transgene copy number (Figure 2A) to analyze siRNA accumulation by high resolution Northern blots.

As demonstrated in Figure 2B, the band intensity of the 22 nt migrating siRNAs (these correspond to the classical 23 nt siRNAs characterized by deep sequencing [15,17,26]) becomes stronger at high temperatures especially for the high copy number line pTI-1. This is apparent for vector specific siRNAs as well as *ND169* specific ones. For the low copy number line pTI-2, Northern blots indicate appearance of the 22 nt band at 31 ${}^{\circ }$C along with a loss of the 1 nt background ladder apparent in the 18 ${}^{\circ }$C and 26 ${}^{\circ }$C lanes. As this background is also apparent for the pTI-1 line, this seems likely due to degradation of the over-expressed aberrant *ND169* transgene RNA. Vice versa, siRNA accumulation appears to be temperature specific at least for the Northern blot detectable 1${}^{\circ }$ transgene siRNAs. The altered ratio between background degradation and specific siRNAs let us hypothesize that altered activity of components of the RNAi machinery are responsible for the temperature effect.

### 3.3. Short Interfering RNA Sequencing Shows Primary and Secondary Short Interfering RNA Temperature Dependency

**Figure 3:**
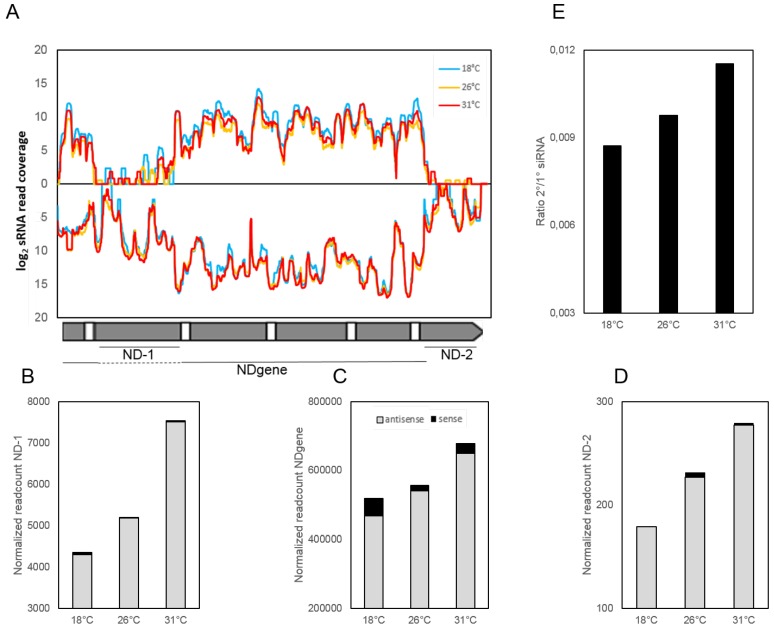
Temperature dependency of transgene associated siRNA accumulation. (**A**) coverage plot of normalized siRNA reads, scaled to a sequencing depth of ten million reads. The log_2_ coverage at different temperatures (18 ${}^{\circ }$C in light blue, 26 ${}^{\circ }$C in orange and 31 ${}^{\circ }$C in red) across the pTI-/- construct is shown. Sense (topside) and antisense (bottom side) reads are separated; (**B**–**D**) quantitative analysis of normalized reads mapping to the ND-1 region (**B**), the NDgene region (**C**) and the ND-2 region (**D**) (antisense/grey and sense/black); (**E**) shows the ratio of total reads of 2${}^{\circ }$ siRNAs (ND-1 and ND-2 region) compared to the amount of reads of 1${}^{\circ }$ siRNAs (NDgene).

Previous studies demonstrated that the amount of 1${}^{\circ }$ siRNAs correlates with the phenotype and predominantly depends on the copy number of the injected transgene [17]. Combining the two factors of copy number and phenotype, different copy number lines of the pTI-/- transgene were analyzed for their phenotype. Supplementary Figure S1 shows that we observe a temperature dependency of the phenotype independent of the copy number because all three different pTI-/- transgene lines show more efficient silencing at 31 ${}^{\circ }$C. To gain more insight into the siRNA characteristics, siRNA samples from the three different temperatures of the high copy number pTI-/- line were sequenced and mapped to the *ND169* gene, thus being able to differentiate between siRNAs of the NDgene region, being predominantly 1${}^{\circ }$ siRNAs and those mapping to the parts missing in the transgene sequence, thus presenting 2${}^{\circ }$ siRNA. Supplementary Figure S2 also shows that, for all analyzed samples and regions, siRNAs are predominantly 23 nt in length; for the 18 ${}^{\circ }$C sample, also many more reads at different read lengths can be observed, which may be due to degradation similar to what we observed in Northern blots of Figure 2. Analyzing siRNAs in coverage blots, Figure 3A shows small RNAs normalized to total reads and mapped to the *ND169* gene indicating a very similar pattern (see Supplementary Figure S3 for the coverage blot with linear axis). We compared reads normalized to the total of macronucleus mapping reads (see Methods) in a better quantitative manner. Figure 3B–D show that 1${}^{\circ }$ as well as 2${}^{\circ }$ siRNA levels increase with temperature; thus, the temperature effect does not solely contribute to 1${}^{\circ }$ siRNA accumulation. In addition, the ratio of both does not appear to be constant as Figure 3E shows that, with increasing temperatures, more 2${}^{\circ }$ relative to 1${}^{\circ }$ siRNAs accumulate. However, we cannot conclude whether this is the result or the reason for higher silencing efficiency.

### 3.4. Increased Short Interfering RNA Accumulation Is Not Due to Increased Precursor RNA Levels

**Figure 4:**
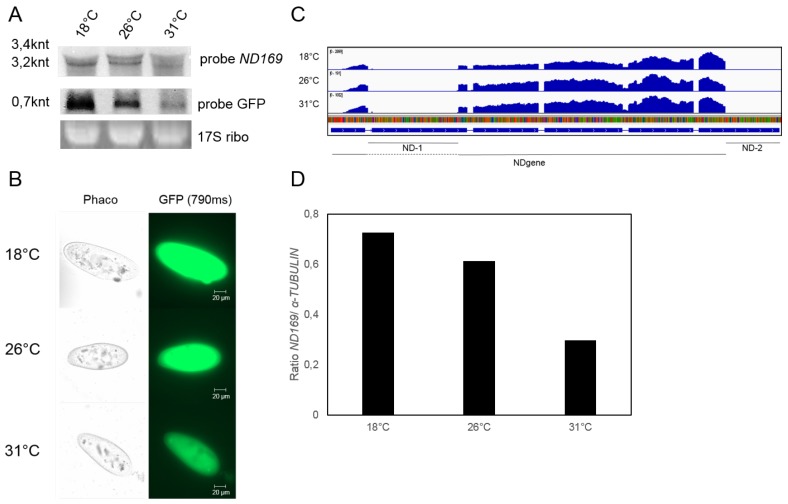
Quantification of precursor RNA levels. (**A**) Northern Blot analysis of *ND169* (top section) and *GFP* (middle section) RNA levels of pTI- cultures at different temperatures (18 ${}^{\circ }$C, 26 ${}^{\circ }$C and 31 ${}^{\circ }$C). 17S ribosomal RNA serves as a loading control (bottom section); (**B**) analysis of the GFP-Expression in cells of a single transgenic line cultivated at the indicated temperatures. The GFP signals of the cells (right, 790 ms exposition) and the same cell seen in phase contrast (left) are shown; (**C**) Integrative Genomics Viewer snapshot of sequencing coverage of the *ND169* gene region with coverage tracks of a pTI-/- line cultivated at different temperatures. The *ND169* gene structure is shown below; (**D**) ratio of *ND169* derived precursor RNA single base coverage from RNAseq-Data, normalized against the single base coverage of the housekeeping gene *α-TUBULIN* at the indicated temperatures.

To determine whether increased levels of 1${}^{\circ }$ short interfering RNAs are due to higher precursor RNA abundance, total RNA from different temperatures was subjected to Northern blots and sequencing. Figure 4A shows a Northern blot hybridized with *ND169* and *GFP* specific probes. We detect a transcript of approx. 3.2 knt (the distance of the promoter to the linearization site) with the *ND169* probe showing decreasing intensity with increasing temperature (endogenous *ND169* mRNA at approx 1.8 knt is usually not detectable in Northern blots). The same is apparent for the *GFP* mRNA. Although we cannot exclude that the latter is due to post-transcriptional inactivation or sensitivity of the *GFP* mRNA, we can confirm this in fluorescence analysis revealing decreasing intensity with increasing temperature (Figure 4B). Decreasing levels of *ND169* transgene RNA can also be confirmed by sequencing. Figure 4C shows coverage blots of un-normalized data indicating no differences in splice efficiency in reads predominantly deriving from the transgene (NDgene region). Normalizing the *ND169* coverage to tubulin, an approximately two-fold decrease can be observed at 31 ${}^{\circ }$C (Figure 4D) consistent with Northern blot analysis.

**Figure 5:**
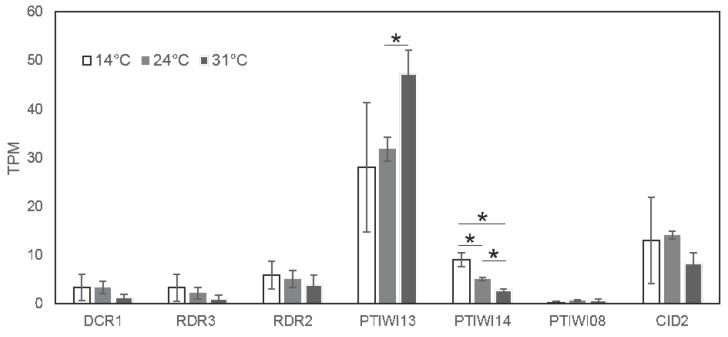
Expression levels of known RNAi components involved in transgene-induced silencing at different temperatures (14 ${}^{\circ }$C, 24 ${}^{\circ }$C or 31 ${}^{\circ }$C). Data is shown in average and standard deviation of three biological replicate TPM values [19]. Significant differences are denoted by * (*p* value < 0.05, Student’s *t*-test).

In summary, these analyses indicate that both transcripts driven by the bidirectional promoter show temperature sensitivity, but our analyses do not allow conclusions whether this is due to transcriptional- or post-transcriptional regulation. However, we can conclude that increased siRNA accumulation is not the result of increased precursor abundance. More likely, the RNAi machinery involved in this particular pathway appears to work more efficiently at higher temperatures, thus leading to more efficient *trans* silencing from the very same amount of injected transgene. The relatively increased 2${}^{\circ }$ siRNA abundance supports the conclusion of a more efficient siRNA accumulation pathway. Taking advantage of a transcriptome resource of *P. tetraurelia*, Figure 5 shows temperature dependent expression data of the known RNAi components involved in transgene induced silencing resulting from poly-A enriched RNAseq of vegetative paramecia [19]. These data indicate that most of the components show surprisingly slightly lower mRNA steady state levels at higher temperatures (*DCR1, RDR3, RDR2, PTIWI14, CID2*). The only component showing temperature dependent induction appears to be *PTIWI13*. As this effect appears to be quite strong, *PTIWI14*, vice versa, shows the strongest reduction. Although we still do not know why two PIWI proteins are necessary for this pathway, their ratio seems to be inversely correlated in a temperature dependent manner. Future studies will need to clarify the contribution of these two PIWIs to temperature dependent *trans* acting siRNA accumulation.

## 4. Discussion

In this report, we describe that a known pathway for epigenetic interaction between two distinct genetic loci in *P. tetraurelia* depends on the environmental temperature. Our data indicates that the efficiency of the silencing phenotype strongly increases with the temperature. This is accompanied by increased accumulation of 1${}^{\circ }$ siRNAs derived from the injected transgene sequence. We therefore conclude that that temperature dependent silencing efficiency is due to altered siRNA biogenesis/accumulation efficiency rather due to altered chromatin remodeling activity or altered precursor transcription. In addition, our data shows that with increasing temperature the relative proportion of 2${}^{\circ }$ siRNAs also increases, which fosters our conclusion that altered siRNA accumulation criteria are involved in this temperature effect. Our current data comprises steady state analyses and we cannot entirely rule out the possibility that other factors e.g., nuclear export of precursor RNA or transcriptional regulation of the transgene RNA could be temperature dependent parameters. However, loss of unspecific degradation products of transgene RNA (vector and *ND169*) with increasing temperatures supports our conclusion of altered siRNA biogenesis. Until now, the individual role of the two distinct RDRs and PIWI proteins involved in transgene induced silencing remains unclear [17]. However, our finding that at least the two PIWIs show inverse temperature dependent expression is consistent with the above hypothesis.

Stress related dependency of siRNA interacting alleles was reported for plants and nematodes. Nuclear RNAi in nematodes, for instance, represses heat stress activation of genes at the transcriptional level, thus buffering the transcriptome against environmental alterations and, as heat stress activation of such genes is heritable in nuclear RNAi mutants, also protecting sexual progeny [27]. In yeast, heat stress has been shown to free Dicer from inactive cytoplasmic aggregates, which then re-localizes into the nucleus repressing the activation of stress genes, thus also representing one more example of epigenetic robustness involving a negative feedback loop [28]. However, these reports of stress induced effects are different to what we observe here. Cultures described in this study were long time adapted to the different cultivation temperatures for at least five days and did not undergo heat stress. A previous transcriptome analysis of cultures cultivated at the same temperatures did not reveal an activation of the known heat shock proteins at 31 ${}^{\circ }$C [19]. Our data indicates a gradual increase of transgene siRNA rather than stress related accumulation. Such a behavior was reported in a similar manner for RNAi mediated virus defense and transgene silencing in *Nicotiana benthamiana* [29]. It has been shown in *A. thaliana* that these two pathways are indeed similar, but differ in the RDR involved [30,31]. The opposite, temperature insensitivity, was, however, reported for perennial grapevines, suggesting that the individual RNAi pathway evolved efficiency/activity according to the life strategy of species because grapevines also need to guarantee cell metabolism and virus defense at low temperatures in contrast to herbaceous plants [32]. In this context, the broad range of temperatures that paramecia can adapt to would be an argument against optimizing RNAi to individual temperatures. We can only speculate here that *P. tetraurelia* could adapt its RNAi machinery to different environments as also suggested by the differential expression of PIWI proteins discussed above. One may also take into account that this ciliates genome consists of a broad variety of individual RNAi components such as as eight Dicers/Dicer-like, four RDRs and 17 PIWI proteins [15,26,33,34]. We therefore need further investigations as to whether diversification and functional specialization of individual RNAi components goes along with differential expression and therefore differential assembly of, for instance, RDRC or PIWI complexes, thus generating functional adapted RNAi complexes depending on environmental circumstances. A recent analysis of endogenous siRNA clusters and their variation along the temperature gradient also supports this idea as we see alternating siRNA accumulation from approx. 1.000 expressed genes (Simon/Schulz, submitted). Thus, the transgene data of this study may be applied to many endogenous loci as well. Further biochemical analyses are required to solve the high complex formation of different RNAi machineries in this model system to study RNAi based transcriptome plasticity.

## Supplementary Materials

The following are available online at http://www.mdpi.com/2073-4425/9/2/117/s1, Figure S1: Silencing phenotypes of three different pTI-/- lines with low (A), medium (B) and high (C) transgene copy number at different temperatures. The percentage of cells categorized by Trich-, Trich+/- and Trich+ after exposure to picric acid is shown. Figure S2: Read length distribution of small RNA reads from different cultivation temperatures mapping to individual regions of the transgene (NDgene) or the endogenous *ND169* gene (ND-1, ND-2). Sense reads are indicated green, antisense reads in orange) Figure S3: Coverage plot of normalized siRNA reads, scaled to a sequencing depth of ten million reads. The linear coverage (18 ${}^{\circ }$C in light blue, 26 ${}^{\circ }$C in orange and 31 ${}^{\circ }$C in red) across the pTI-/- construct is shown. Sense and antisense reads are separated.
