# Supplementary material for: Environmental Temperature Controls Accumulation of Transacting siRNAs Involved in Heterochromatin Formation
Source: Genes (Basel). 2018 Feb 21;9(2):117. doi: 10.3390/genes9020117 (PMC5852613; doi:10.3390/genes9020117)
Supplement: Supplementary file 1 [file genes-09-00117-s001.zip › genes-264579/logo-mdpi-eps-converted-to.pdf]

MDPI
