# Supplementary material for: Environmental Temperature Controls Accumulation of Transacting siRNAs Involved in Heterochromatin Formation
Source: Genes (Basel). 2018 Feb 21;9(2):117. doi: 10.3390/genes9020117 (PMC5852613; doi:10.3390/genes9020117)
Supplement: Supplementary file 1 [file genes-09-00117-s001.zip › genes-264579/template.pdf]

Article

Environmental temperature controls accumulation of transacting siRNAs involved in heterochromatin formation

Marcello Pirritano [1,‡], Ulrike Götz [1,‡], Sivarajan Karunanithi [3], Karl Nordström [2], Marcel H. Schulz [3] and Martin Simon [1*]

1 Molecular Cell Dynamics, Centre for Human and Molecular Biology; Saarland University; martin.simon@uni-saarland.de
2 Genetics/Epigenetics, Centre for Human and Molecular Biology, Saarland University; karl.nordstroem@uni-saarland.de
3 Cluster of Excellence, Multimodal Computing and Interaction, Saarland University and Department for Computational Biology and Applied Algorithmics, Max Planck Institute for Informatics, Saarland Informatics Campus ; mschulz@mmci.uni-saarland.de
* Correspondence: martin.simon@uni-saarland.de; Tel.: +49-691-302-4938
‡ These authors contributed equally to this work.

Academic Editor: name
Version January 4, 2018 submitted to Genes; Typeset by LaTeX using class file mdpi.cls

Abstract: Genes or alleles can interact by small RNAs in a homology dependent manner meaning that siRNAs can act *in trans* at the chromatin level producing stable and heritable silencing phenotypes. Because of the puzzling data on endogenous paramutations, their impact contributing to adaptive evolution in a Lamarckian manner remains unknown. An increasing number of studies characterizes the underlying siRNA accumulation pathways using transgene experiments. Also in the ciliate *Paramecium*, we induce *trans* silencing on the chromatin level by injection of truncated, non-expressible transgenes. Here, we characterize the efficiency of this mechanism at different temperatures showing that silencing of the endogenous genes is temperature dependent. Analyzing different transgene constructs at different copy numbers, we dissected whether silencing efficiency is due to varying precursor RNAs or siRNA accumulation. Our data shows that silencing efficiency correlates with more efficient accumulation of primary siRNAs at higher temperatures rather than higher expression of precursor RNAs by the triggering transgene. Due to higher primary levels, also secondary siRNAs show temperature dependency and interestingly also increase their relative proportion to primary siRNAs. Our data shows that efficient *trans* silencing on the chromatin level in *Paramecium* depends on environmental parameters thus being an important epigenetic factor limiting regulatory effects of siRNAs.

Keywords: RNA interference; transitivity; chromatin; environment

---

1. Introduction

Next to the post-transcriptional inactivation of gene expression by siRNAs (short interfering) or miRNAs (microRNAs), epigenetic silencing of genes occurs on the chromatin level involving siRNAs targeting loci for histone modifications in a homology dependent manner. Strikingly, this kind of co-transcriptional silencing represents a self enforcing feedback mechanism thus enabling self replicating epigenetic states [33]. Also trans-generational epigenetic gene silencing by small RNAs has been shown in many model systems ranging from ciliates to plants and animals although such mechanisms in mammals are still under debate (reviewed in [34,35]). However, heritable epigenetic silencing could be a powerful adaptation processes in a Lamarckian manner.

The ability of siRNAs to act *in trans* to another allele or homologous locus appears to be restricted. In *Schizosaccharomyces pombe* for instance, gene position effects as well as siRNA abundance are important factors [36,37]. Therefore, many individual parameters need to be taken into account whether siRNAs can act *in trans* or not. Next to the individual characteristics of a specific pathway including its genetic requirements and the amount of precursor produced, also environmental issues need to be considered. Paramutations, meaning the heritable silencing of homologous alleles in plants for instance are quantitative events being sensitive to environmental stressors or growth conditions [38]. Also in animals (*Drosophila melanogaster* and *Caenorhabditis elegans*), *trans* silencing by siRNAs in a paramutative manner has been observed [39,40]. Although *trans* silencing and paramutation can be followed in *C. elegans* using different exogenic reporters, many endogenous genes are protected from silencing although a subset of genes indeed follows the behavior of the exogenic transgene reporters [39]. Although these findings tempt speculation on the evolutionary impact of this kind of quantitative epigenetics, the molecular basis of quantitative silencing is hardly understood in this context. As induced epigenetic modifications and their potential trans-generational persistence cannot be a general genome wide phenomenon rather than limited to individual plastic genes, we need to understand what makes genes sensitive for *trans* silencing thus discriminating between epigenetic stability or variability [41].

Paramecium is a unicellular model in genetics and epigenetics [42]. RNAi can be triggered by feeding of dsRNA producing bacteria similar to *C. elegans* [43] or by injection of truncated transgenes [44–46]. Both pathways apparently differ in their siRNAs and genetic requirements although individual components such as *DCR1* and *RDR2* are shared [47–49]. Silencing endogenous genes by injection transgenes producing incomplete mRNA triggers accumulation of 23nt siRNAs. These primary (1°) siRNAs can act *in trans* to endogenous remote loci triggering loss of activating H3K4me3 and H3K9ac and accumulation of H3K27me3. Similar to the mechanism of co-transcriptional silencing , the remote locus then produces secondary (2°) siRNAs with strong antisense bias and 23nt lenght with decreasing coverage from the 5'- to the 3'- end of the open reading frame [49]. We show in this paper, that this process depends on the environmental temperature. In contrast to homoiothermic animals, *Paramecium* is a poikilothermic species. Thus, this single cell has to guaranty that all metabolic pathways run properly at the environmental temperature similar to the problems also plants and several homoiothermic animals face with. It has been known for a long time, that paramecia tolerate a broad spectrum of temperatures ranging from 4°C to almost 37°C for stable cultivation [50,51]. Our data here indicates that the RNAi machinery rather than the transcription of a differential precursor is responsible for differential siRNA accumulation at different environmental temperatures.

2. Results

2.1. Silencing phenotypes by truncated transgenes depend on the environmental temperature

As transgenes injected into the somatic macronucleus are lost when cells start undergoing autogamy or conjugation, the transgene is maintained only in a single generation. Thus, after injection of young cells, transgene cultures are usually maintained in stocks at low temperatures (4°C-6°C) to limit cell divisions which is the main factor defining clonal age for ciliates (reviewed in [52]). When going back to stock cultures of constructs which induce homology dependent gene silencing of endogenous remote loci, we realized that these do not show silencing phenotypes rather being wild type at low temperatures. Figure 1A shows plasmid maps of two transgene constructs (pTI- and pTI-/-) containing truncated versions of the *ND169* gene, its gene product necessary for trichocyst ejection. The truncated versions have been shown to accumulate long aberrant RNA triggering siRNA accumulation acting *in trans* to the endogenous *ND169* locus [47,49]. Figure 1B shows that silencing efficiency of the *ND169* gene increases with the temperature in cultures maintained from 18-31°C indicated by higher proportion of cells showing lower degree or no trichocyst discharge as indicated

in Figure 1C. We consequently asked for the reason of the temperature dependency of *trans* silencing in *Paramecium* and subsequently analyzed siRNA accumulation as well as precursor RNA abundance to gain insight into the molecular background.

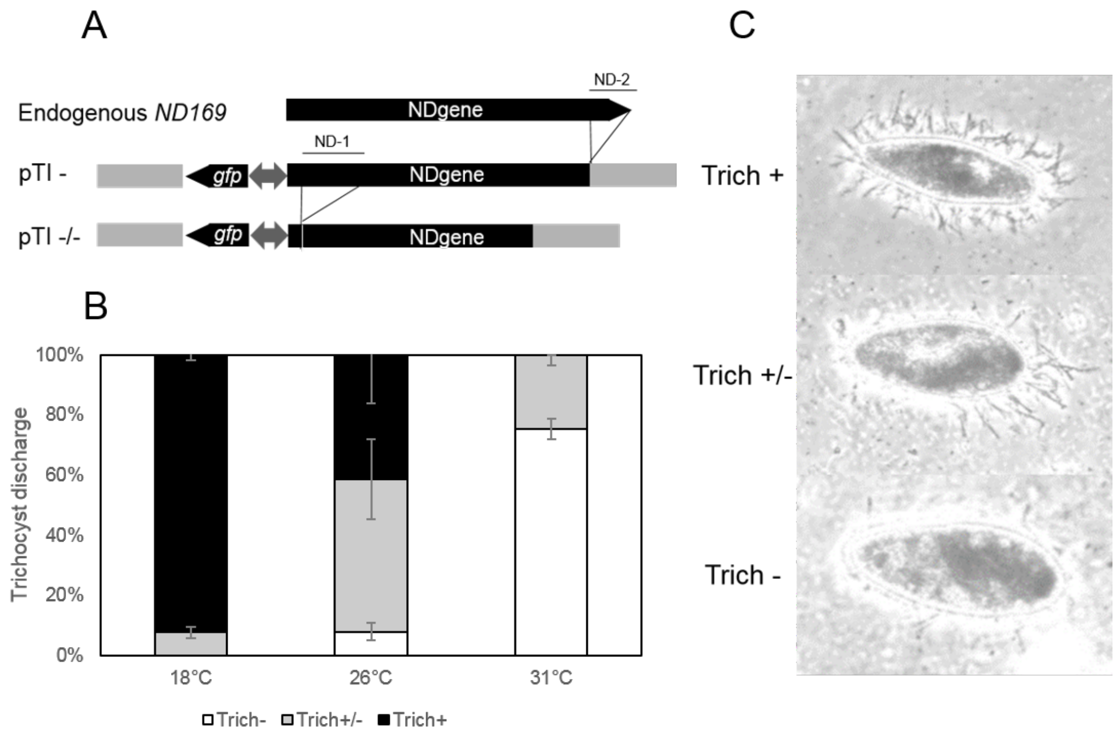

Figure 1. Transgene constructs and phenotypes of transgene-induced silencing. (**A**) Overview of constructs. Shown are the endogenous version of the gene *ND169* (upper part), the pTI- construct, a 3'-coding region truncated version of the *ND169* gene (ND-2) and the pTI-/- construct, with additional deletions of the 5'-coding region (ND-1). Both constructs also contain a *gfp* gene under control of a bidirectional promoter. (**B**) Quantification of trichocyst discharge phenotypes in pTI-/- lines at the indicated temperatures. Shown are percentages of cells with different categories of trichocyst ejection, ranging between a complete loss of gene expression (Trich-), an intermediate phenotype (Trich+/-) and no silencing phenotype at all (Trich+). Standard deviation and proportions of the different phenotypes are means of three biological replicates, respectively. (**C**) Example of the observed trichocyst phenotypes. Phase contrast pictures show WT cells with full trichocyst discharge (upper panel, Trich+), a partial phenotype with few visible trichocysts (middle panel, Teich +/-) and cells showing efficient silencing and no trichocyst discharge after treatment with picric acid (lower panel, Trich-).

2.2. Cold temperatures impede siRNA accumulation

We first asked for the accumulation of siRNAs. Molecular analysis was carried out for two transgenic pTI- lines with either low or high transgene copy number (Figure 2A) to analyze siRNA accumulation by high resolution Northern blots. As demonstrated in Figure 2B, the band intensity of the 22nt migrating siRNAs (these correspond to the classical 23nt siRNAs characterized by deep sequencing [47,49,53]) becomes stronger at high temperatures especially for the high copy number line pTI-1. This is apparent for vector specific siRNAs as well as *ND169* specific ones. For the low copy number line pTI-2, Northerns indicate appearance of the 22nt band at 31°C along with a loss of the 1nt background ladder apparent in the 18°C and 26°C lanes. As this background is also apparent for the pTI-1 line, this seems likely due to degradation of the over-expressed aberrant *ND169* transgene RNA. Vice versa, siRNA accumulation appears to be temperature specific at least for the Northern

detectable 1° transgene siRNAs. The altered ratio between background degradation and specific siRNAs let us hypothesize that altered activity of components of the RNAi machinery are responsible for the temperature effect.

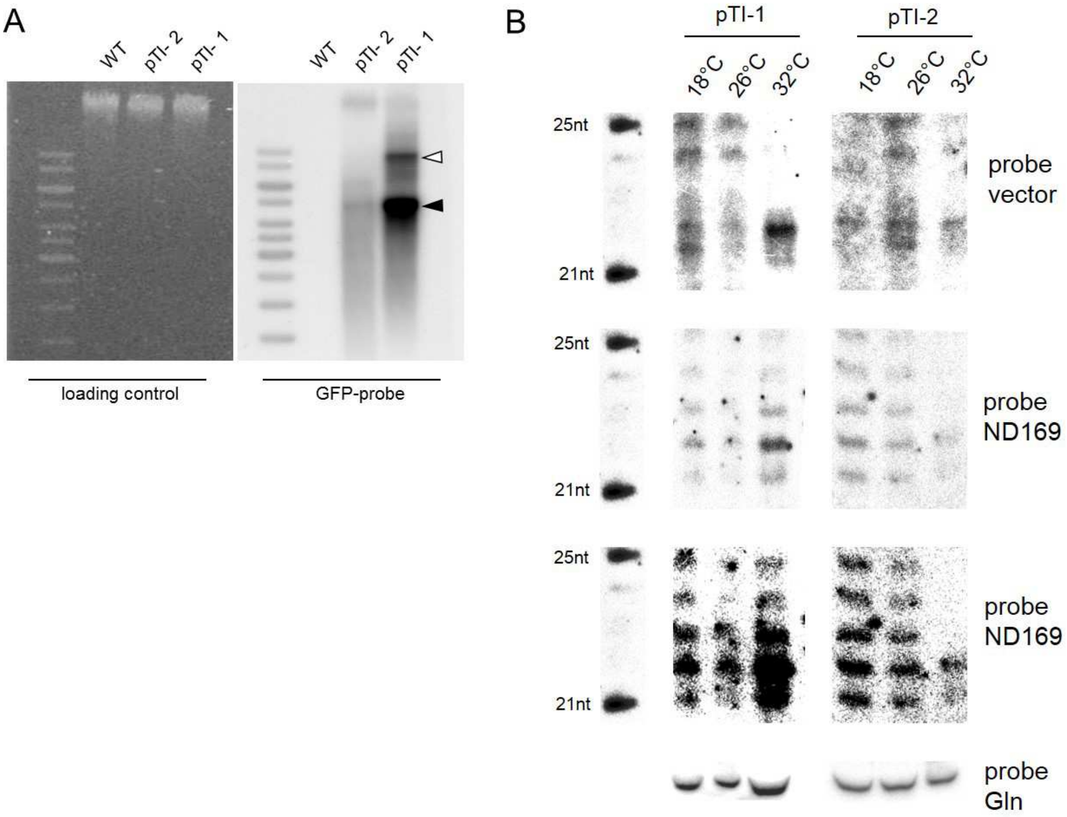

Figure 2. Analysis of temperature dependent siRNAs in low and high copy number injected transgenic lines. (**A**) Southern blot of macronuclear DNA isolated from WT and two pTI- lines (pTI-1, pTI-2) hybridized with a *GFP* specific probe (right). The ethidium bromide stained gel before blotting is shown on the left. The black arrowhead points to transgene mini-chromosomes in the size of the linearized plasmid of approx. 4475bp with additional telomers. The open arrowhead points to transgene dimers resulting from endogenous end repair. (**B**) Northern blot of siRNAs of the two transgene lines cultivated at the indicated temperatures. The marker is shown on the left (5'-OH), a probe against the Gln tRNA serves as a loading control. The upper panel shows hybridizations with a vector specific probe, downstream of the truncated *ND169* gene region. Beneath, hybridizations with a *ND169* specific probe are shown, in the middle with low, in the lower panel with longer exposure.

2.3. siRNA sequencing shows primary and secondary siRNA temperature dependency

Previous studies demonstrated that the amount of 1° siRNAs correlates with the phenotype and predominantly depends on the copy number of the injected transgene [49]. Combining the two factors of copy number and phenotype, different copy number lines of the pTI-/- were analyzed for their phenotype at different temperatures. Supplementary Figure S1 shows that we observe a general temperature dependency of the phenotype independent of the copy number meaning the three different pTI-/- transgene lines show more efficient silencing at 31°C although the overall efficiency remain still higher in the high copy number transgene line. To gain more insight into the siRNA characteristics, siRNA samples from the three different temperatures of the high copy number pTI-/- line were sequenced and mapped to the *ND169* gene thus being able to differentiate between siRNAs of the NDgene region, being predominantly 1° siRNAs and those mapping to the parts missing in the transgene sequence thus presenting 2° siRNA. Supplementary Figure S2 also shows that for all

analyzed samples and regions, siRNAs are predominantly 23nt in length; for the 18°C sample also many more reads at different read lengths can be observed which may be due to degradation similar to what we observed in Northerns of Figure 2. Analyzing siRNAs in coverage blots, Figure 3A shows small RNAs normalized to total reads and mapped to the *ND169* gene indicating a very similar pattern. As the log scale here hides many differences between the individual lines, we analyzing reads normalized to the total of macronucleus mapping reads (see Methods) in a better quantitative manner. Figure 3B-D show that 1° as well as 2° siRNA levels increase with temperature, thus the temperature effect does not solely contribute to 1° siRNA accumulation. In addition, the ratio of both does not appear constant as Figure 3E shows that with increasing temperatures more 2° relative to 1° siRNAs accumulate. However, we cannot conclude whether this is the result or the reason for higher silencing efficiency.

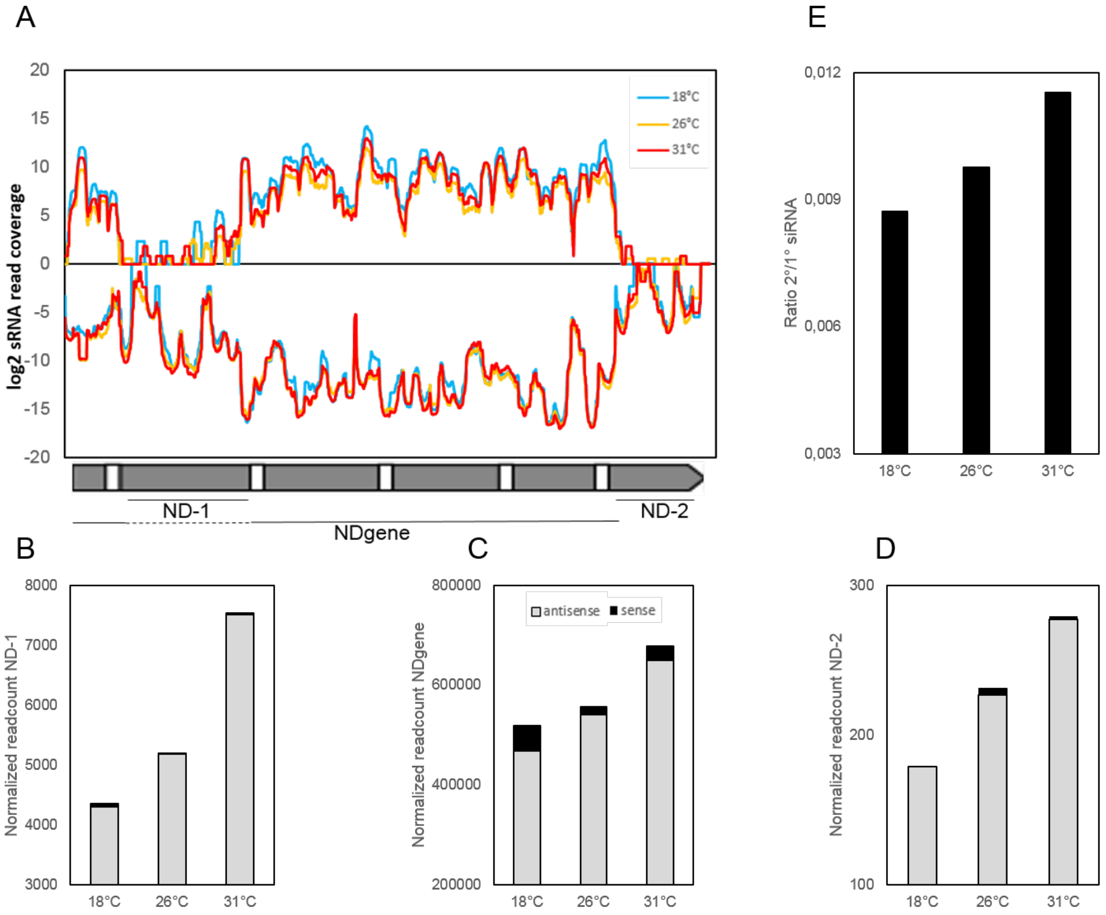

Figure 3. Temperature dependency of transgene associated siRNA accumulation. (**A**) Coverage plot of normalized siRNA reads, scaled to a sequencing depth of ten million reads. The log2 coverage at different temperatures (18°C in light blue, 26°C in orange and 32°C in red) across the pTI-/- construct is shown. Sense (positive) and antisense (negative) reads are separated. (**B**,**C**,**D**) Quantitative analysis of normalized reads mapping to the ND-1 region (**B**), the NDgene region (**C**) and the ND-2 region (**D**) (antisense/grey and sense/black). (**E**) shows the ratio of total reads of 2° siRNAs (ND-1 and ND-2 region) compared to the amount of reads of 1° siRNAs (NDgene) at the different temperatures.

2.4. Increased siRNA accumulation is not due to increased precursor RNA levels

To determine whether increased levels of 1°siRNAs are due to higher precursor RNA abundance, total RNA from different temperatures was subjected to Northern blots and sequencing. Figure 4A shows a Northern blot hybridized with probes against *ND169* and *GFP*. We detect a transcript

of approx. 3.2kb (the distance of the promoter to the linearization site) with the *ND169* probe showing decreasing intensity with increasing temperature. The same is true for the *GFP* mRNA. Although we cannot exclude that the latter is due to post-transcriptional inactivation or sensitivity of the *GFP* mRNA, we can confirm this in fluorescence analysis revealing decreasing intensity with increasing temperature (Figure 4B). Decreasing levels of *ND169* transgene RNA can also be confirmed by sequencing. Figure 4C shows coverage blots of unnormalized data indicating no differences in splice efficiency. Normalizing the *ND169* coverage to tubulin, an approx. two fold decrease can be observed at 31°C (Figure 4D) consistent with Northern analysis.

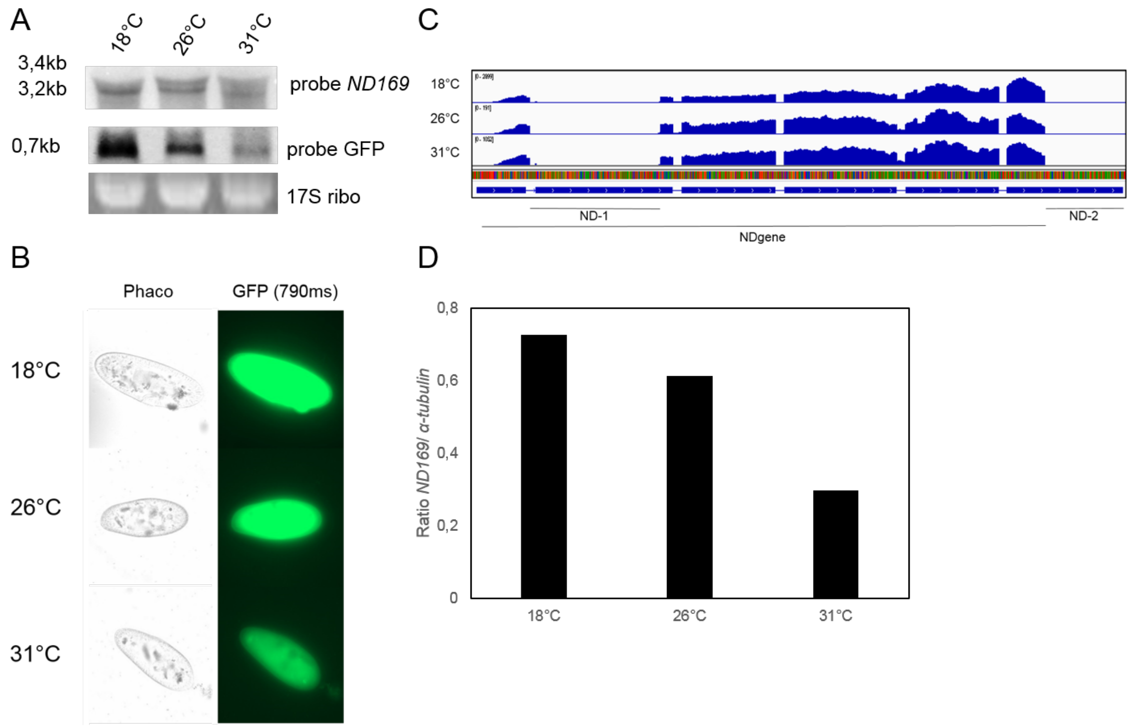

Figure 4. Quantification of precursor RNA levels. (**A**) Northern Blot analysis of *ND169* (top section) and *gfp* (middle section) mRNA levels of pTI- cultures at different temperatures (18°C, 26°C and 31°C). 17S ribosomal RNA serves as a loading control (bottom section). (**B**) Analysis of the GFP-Expression in cells of a single transgenic line cultivated at the indicated temperatures. Shown are the GFP signals of the cells (right, 790ms exposition) and the same cell seen in phase contrast (left). (**C**) IGV snapshot of sequencing coverage of the *ND169* gene region. Shown are the coverage tracks of the NGS sequencing of a single pTI-/- line, cultivated at different temperatures. The *ND169* gene structure as well as the observed regions (ND-1, ND-2 and NDgene) are displayed below. (**D**) Ratio of *ND169* derived precursor RNA single base coverage from RNAseq-Data, normalized against the single base coverage of the housekeeping gene α-tubulin at the indicated temperatures.

In summary, these analyses indicate that both transcripts driven by the bidirectional promoter show temperature sensitivity but our analyses do not allow conclusions whether this is due to transcriptional- or post-transcriptional regulation. However, we can conclude that increased siRNA accumulation is not the result of increased precursor abundance. More likely, the RNA machinery involved in this particular pathway appears to work more efficient at higher temperatures thus leading to more efficient *trans* silencing from the very same amount of injected transgene. The relatively increased 2° siRNA abundance supports the conclusion of a more efficient siRNA accumulation pathway.

Figure 5 shows temperature dependent expression data of the known RNAi components involved in transgene induced silencing. These indicate that most of the components show

surprisingly slightly lower mRNA steady state levels at higher temperatures (*DCR1, RDR3, RDR2, PTIWI14, CID2*). The only component showing temperature dependent induction appears to be *PTIWI13*. As this effect appears to be quite strong, *PTIWI14* vice versa shows the strongest reduction. Although we still do not know why two PIWI proteins are necessary for this pathway, their ratio seems to be inversely correlated in a temperature dependent manner. Future studies will need to clarify the contribution of these two PIWIs to temperature dependent *trans* acting siRNA accumulation.

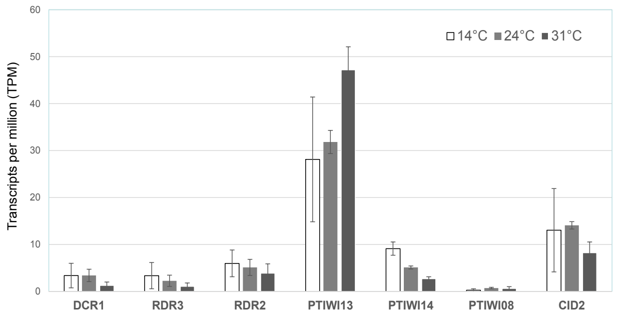

Figure 5. Expression levels of known RNAi components involved in transgene-induced silencing at different temperatures (14°C, 24°C or 31°C). Data is shown in average and standard deviation of three biological replicate TPM values[51].

3. Discussion

In this report, we describe that a known pathway for epigenetic interaction between two distinct genetic loci in *Paramecium* depends in the environmental temperature. Our data indicates that the efficiency of the silencing phenotype strongly increases with the temperature with a maximum knock down at 31°C. This is accompanied by increased accumulation of 1° siRNAs derived form the injected transgene sequence. We therefore conclude that that temperature dependent silencing efficiency is due to altered siRNA biogenesis/accumulation efficiency rather due to altered chromatin remodeling activity or altered precursor transcription. In addition, our data shows that with increasing temperature the relative proportion of 2° siRNAs also increases which fosters our conclusion that altered siRNA accumulation criteria are involved in this temperature effect. The loss of unspecific degradation products of transgene RNA (Vector and *ND169*) with increasing temperatures supports this idea. Until now, the individual role of the two distinct RDRs and PIWI proteins involved in transgene induced silencing remains unclear [49]. However, our finding that at least the two PIWIs show inverse temperature dependent expression is consistent with the above hypothesis.

Stress related dependency of siRNA interacting alleles was reported for plants and nematodes. Nuclear RNAi in nematodes for instance represses heat stress activation of genes at the transcriptional level thus buffering the transcriptome against environmental alterations and, as heat stress activation of such genes is heritable in nuclear RNAi mutants, also protecting sexual progeny [54]. In yeast, heat stress has been shown to free Dicer from inactive cytoplasmic aggregates which then re-localizes into the nucleus repressing the activation of stress genes thus also representing one more example of epigenetic robustness involving a negative feedback loop [55].

However, these reports of stress induced effects are different to what we observe here. Cultures described in this study were long time adapted to the different cultivation temperatures for at least five days and did not undergo heat stress. A previous transcriptome analysis of cultures cultivated at the same temperatures did not reveal an activation of the known heat shock proteins at 31°C [56]. Our data indicates a gradual increase of transgene siRNA rather than stress related accumulation. Such a behavior was reported in a similar manner for RNAi mediated virus defense and transgene silencing in *Nicotiana benthamiana* [57]. It has been shown in *Arabidopsis* that these two pathways are indeed similar, but differ in the RDR which is involved [58,59]. The opposite, temperature in-sensitivity, was

however reported for perennial grapevines, suggesting that the individual RNAi pathway evolved efficiency/activity according to the life strategy of species because grapevines also need to guaranty cell metabolism and virus defense at low temperatures in contrast to herbaceous plants [60]. In this context, the broad range of temperatures paramecia can adapt to would be an argument against optimizing RNAi to individual temperatures. We can only speculate here that *Paramecium* could adapt its RNAi machinery to different environments as also suggested by the differential expression of PIWI proteins discussed above. One may also take into account that this ciliates genome consists of a broad variety of individual RNAi components such as as eight Dicers/Dicer-like, four RDRs and 17 PIWI proteins [53,61–63]. We need therefore further investigations whether diversification and functional specialization of individual RNAi components goes along with differential expression and therefore differential assembly of for instance RDRC or PIWI complexes thus generating functional adapted RNAi complexes depending on environmental circumstances. A recent analysis of endogenous siRNA clusters and their variation along the temperature gradient also supports this idea as we see alternating siRNA accumulation from approx. 1500 expressed genes (Simon/Schulz, submitted) suggesting that transgene data of this study may be applied to many endogenous loci as well. Further biochemical analyses are required to solve the high complex formation of different RNAi machineries in this model system to study RNAi based transcriptome plasticity.

4. Materials and Methods

- Cell culture, transgenic lines and phenotypic characterization

Paramecium tetraurelia strain 51 was cultivated in wheat grass powder medium (WGP, Pines International Co., Lawrence, KS) supplemented with 0,8 μg/ml β-sitosterol and inoculated with *Klebsiella pneumonia* as food bacteria. Transformation of single cells was performed by using microinjection as described before [47]. The pTI- and pTI-/- constructs containing the open reading frame of *gfp* and a truncated version of the *ND169* gene under control of a bidirectional promoter used for microinjection were described in [49].

Successfully transfected cells were validated by GFP expression and cultivated at 4°C to build up a stock and prevent autogamy. Cells of a transgenic line were split, slowly adapted to the indicated experimental temperature (18°C, 26°C, 31°C) and cultivated until the necessary cell number for DNA or RNA extraction was reached. While cultivating at the different experimental temperatures, the efficiency of the *ND169* gene silencing triggered by the transfected construct was observed by trichocyst discharge using picric acid.

- DNA isolation and Southern blots

Intact macronuclear chromosomes were isolated by lysing cells in 0.5M EDTA pH 9; 1% sarcosyl; 1% SDS; 0.25mg/ml Proteinase K and incubation at 55°C over night. After phenol extraction and dialysis against TE buffer for 48hours , the DNA was digested with RNAse A and additionally extracted with phenol. Agarose gel electrophoresis and Southern blots were carried out by standard capillary procedures including depurination .

- Total RNA isolation and Northern blots

Total RNA from 50.000 *Paramecium tetraurelia* cells was isolated using TriReagent® (Sigma–Aldrich, Seelze, Germany). Integrity was analyzed by denaturating gels or Agilent Bioanalyzer Chips. For standard Northern blots, gel electrophoresis of 15μg total RNA was performed using a formaldehyde denaturating 1,2% agarose gel. Afterwards, the separated RNA was blotted in 10xSSC to a Hybond N+ membranes (GE/Amersham, Braunschweig, Germany) using standard capillary northern procedures. Crosslinking of the blotted RNA (and DNA of Southerns) to the membranes was performed using 70kJ/cm2UV.

- Non radioactive labeling and hybridizations

Probes for hybridizations to long nucleic acids (*ND169* transgene, *ND169* mRNA, *GFP* mRNA) were generated by PCR products (position 701-1733 for *ND169* and position 12-559 for *eGFP*). Biotin labeling using Klenow exo- and random decamers was carried out using the BiotinDeca Labeling Kit (Thermo Fisher, Darmstadt, Germany). Hybridizations to Northern and Southern blots were carried out at 60°C in Church buffer and signals were visualized by alkaline phosphatase (Biotin Chromogenic detection Kit, Thermo Fisher, Darmstadt, Germany).

- siRNA Northern blot analysis

For smallRNA Northern blots, 50μg total RNA was separated on a 40cm urea polyacrylamid gel (15%, 19:1 acrylamid:bis) and vacuum blotted in 20xSSC to Hybond N + membranes (GE/Amersham, Braunschweig, Germany). Radiolabeling of probes was carried out with [a-32P] dCTP (3000 Ci/mmol) using random octamers and Klenow exo-. Oligos complementary to the marker and Gln tRNA were 5′ -labeled with T4 polynucleotid kinase using [g-32P] ATP (3000 Ci/mmol). Primers for the vector specific probe of the pTi- plasmid from position 817-2543 relative to the *GFP* start codon in the pTI- plasmid.

- siRNA library preparation, Illumina sequencing and bioinformatical analyses

smallRNAs (17-25nt size) from 20μg total RNA were size selected by gel extraction using a 17,5% urea acrylamid mini-gel, visualized with SYBR®-Gold (Life-Technologies, Darmstadt, Germany). Gel slizes containing the wanted RNA-Fraction were cut into small pieces and RNA was eluated by overnight incubation in 3 Vol. 0,3M NaCl at 4°C. RNA was precipitated using 3 Vol. Ethanol (100%), 1/10 Vol. NaAcetat (3M, pH5) and Glycogen (70ng/μl) and afterwards subjected to library preperation using the NEBNext® small RNA library prep Kit (NEB, Frankfurt a.M., Germany), according to the manufacturer's instructions (3'-adapter ligation extended to 18h at 16°C). Sequencing was done on the Illumina HiSEQ 2500 platform using the RAPID mode and 30nt read length. Reads were de-multiplexed and adapter sequences were trimmed using Trim Galore (http://www.bioinformatics.babraham.ac.uk/projects/trim galore/) that uses Cutadapt [64] with a stringency cutoff of 10. smallRNA read alignments to transgenes and normalization of reads was carried out precisely as described in [49]. The complete analysis pipeline is available under https://github.com/SchulzLab/RAPID.

Supplementary Materials: Figure S1: Silencing phenotypes of three different pTI-/- lines with low (A), medium(B) and high (C) transgene copy number at different temperatures. Shown is the percentage of cells categorized by Trich-, Trich+/- and Trich+ after exposure to picric acid. Figure S2: Read length distribution of small RNA reads from different cultivation temperatures mapping to individual regions of the transgene (NDgene) or the endogenous *ND169* gene (ND-1, ND-2). Sense reads are indicated green, antisense reads in orange)

Acknowledgments: This work was supported by grants from the German research Council (DFG) to MS (SI1379/3-1) and to SCHU (3140/1-1). MP receives a undergraduate scholarship by the German Academic Scholarship Foundation.

Author Contributions: MP and UG performed the experiments and analyzed the data. SK and MHS processed and analyzed small RNA data . KN analyzed long RNA data and contributed NGS analysis tools. MS conceived the study and wrote the paper.

Conflicts of Interest: The authors declare no conflict of interest.The founding sponsors had no role in the design of the study; in the collection, analyses, or interpretation of data; in the writing of the manuscript, and in the decision to publish the results'.

Bibliography

33. Holoch, D.; Moazed, D. RNA-mediated epigenetic regulation of gene expression. *Nature Reviews Genetics* **2015**, *16*, 71–84.
34. Neeb, Z.T.; Nowacki, M. RNA-mediated transgenerational inheritance in ciliates and plants. *Chromosoma* **2017**, pp. 1–9.

35. Sarkies, P.; Miska, E.A. Small RNAs break out: the molecular cell biology of mobile small RNAs. *Nature Reviews Molecular Cell Biology* **2014**, *15*, 525–535.
36. Bühler, M.; Verdel, A.; Moazed, D. Tethering RITS to a nascent transcript initiates RNAi-and heterochromatin-dependent gene silencing. *Cell* **2006**, *125*, 873–886.
37. Simmer, F.; Buscaino, A.; Kos-Braun, I.C.; Kagansky, A.; Boukaba, A.; Urano, T.; Kerr, A.R.; Allshire, R.C. Hairpin RNA induces secondary small interfering RNA synthesis and silencing in trans in fission yeast. *EMBO reports* **2010**, *11*, 112–118.
38. Hollick, J.B. Paramutation and related phenomena in diverse species. *Nature reviews Genetics* **2017**, *18*, 5–23.
39. Sapetschnig, A.; Sarkies, P.; Lehrbach, N.J.; Miska, E.A. Tertiary siRNAs mediate paramutation in C. elegans. *PLoS genetics* **2015**, *11*, e1005078.
40. De Vanssay, A.; Bougé, A.L.; Boivin, A.; Hermant, C.; Teysset, L.; Delmarre, V.; Antoniewski, C.; Ronsseray, S. Paramutation in Drosophila linked to emergence of a piRNA-producing locus. *Nature* **2012**, *490*, 112–115.
41. Tricker, P.J. Transgenerational inheritance or resetting of stress-induced epigenetic modifications: two sides of the same coin. *Frontiers in plant science* **2015**, *6*.
42. Simon, M.; Plattner, H. Unicellular eukaryotes as models in cell and molecular biology: critical appraisal of their past and future value. *Int Rev Cell Mol Biol* **2014**, *309*, 141–198.
43. Galvani, A.; Sperling, L. RNA interference by feeding in Paramecium. *TRENDS in Genetics* **2002**, *18*, 11–12.
44. Ruiz, F.; Vayssié, L.; Klotz, C.; Sperling, L.; Madeddu, L. Homology-dependent gene silencing in Paramecium. *Molecular biology of the cell* **1998**, *9*, 931–943.
45. Garnier, O.; Serrano, V.; Duharcourt, S.; Meyer, E. RNA-mediated programming of developmental genome rearrangements in Paramecium tetraurelia. *Molecular and cellular biology* **2004**, *24*, 7370–7379.
46. Galvani, A.; Sperling, L. Transgene-mediated post-transcriptional gene silencing is inhibited by 3 non-coding sequences in Paramecium. *Nucleic acids research* **2001**, *29*, 4387–4394.
47. Marker, S.; Le Mouel, A.; Meyer, E.; Simon, M. Distinct RNA-dependent RNA polymerases are required for RNAi triggered by double-stranded RNA versus truncated transgenes in Paramecium tetraurelia. *Nucleic Acids Res* **2010**, *38*, 4092–107.
48. Marker, S.; Carradec, Q.; Tanty, V.; Arnaiz, O.; Meyer, E. A forward genetic screen reveals essential and non-essential RNAi factors in Paramecium tetraurelia. *Nucleic acids research* **2014**, *42*, 7268–7280.
49. Götz, U.; Marker, S.; Cheaib, M.; Andresen, K.; Shrestha, S.; Durai, D.A.; Nordström, K.J.; Schulz, M.H.; Simon, M. Two sets of RNAi components are required for heterochromatin formation in trans triggered by truncated transgenes. *Nucleic acids research* **2016**, *44*, 5908–5923.
50. Beale, G.H.; others. The genetics of Paramecium aurelia. *The genetics of Paramecium aurelia.* **1954**.
51. Cheaib, M.; Dehghani Amirabad, A.; Nordstrom, K.J.; Schulz, M.H.; Simon, M. Epigenetic regulation of serotype expression antagonizes transcriptome dynamics in Paramecium tetraurelia. *DNA Res* **2015**, *22*, 293–305.
52. Meyer, E.; Chalker, D.L.; others. Epigenetics of ciliates. *Epigenetics* **2007**, pp. 127–150.
53. Lepere, G.; Nowacki, M.; Serrano, V.; Gout, J.F.; Guglielmi, G.; Duharcourt, S.; Meyer, E. Silencing-associated and meiosis-specific small RNA pathways in Paramecium tetraurelia. *Nucleic acids research* **2008**, *37*, 903–915.
54. Ni, J.Z.; Kalinava, N.; Chen, E.; Huang, A.; Trinh, T.; Gu, S.G. A transgenerational role of the germline nuclear RNAi pathway in repressing heat stress-induced transcriptional activation in C. elegans. *Epigenetics & chromatin* **2016**, *9*, 3.
55. Oberti, D.; Biasini, A.; Kirschmann, M.A.; Genoud, C.; Stunnenberg, R.; Shimada, Y.; Bühler, M. Dicer and HSP104 function in a negative feedback loop to confer robustness to environmental stress. *Cell reports* **2015**, *10*, 47–61.
56. Cheaib, M.; Dehghani Amirabad, A.; Nordström, K.J.; Schulz, M.H.; Simon, M. Epigenetic regulation of serotype expression antagonizes transcriptome dynamics in Paramecium tetraurelia. *DNA Research* **2015**, *22*, 293–305.
57. Szittya, G.; Silhavy, D.; Molnár, A.; Havelda, Z.; Lovas, Á.; Lakatos, L.; Bánfalvi, Z.; Burgyán, J. Low temperature inhibits RNA silencing-mediated defence by the control of siRNA generation. *The EMBO Journal* **2003**, *22*, 633–640.

58. Voinnet, O. RNA silencing as a plant immune system against viruses. *TRENDS in Genetics* **2001**, *17*, 449–459.
59. Dalmay, T.; Hamilton, A.; Rudd, S.; Angell, S.; Baulcombe, D.C. An RNA-dependent RNA polymerase gene in Arabidopsis is required for posttranscriptional gene silencing mediated by a transgene but not by a virus. *Cell* **2000**, *101*, 543–553.
60. Romon, M.; Soustre-Gacougnolle, I.; Schmitt, C.; Perrin, M.; Burdloff, Y.; Chevalier, E.; Mutterer, J.; Himber, C.; Zervudacki, J.; Montavon, T.; others. RNA silencing is resistant to low-temperature in grapevine. *PloS one* **2013**, *8*, e82652.
61. Sandoval, P.Y.; Swart, E.C.; Arambasic, M.; Nowacki, M. Functional diversification of Dicer-like proteins and small RNAs required for genome sculpting. *Developmental cell* **2014**, *28*, 174–188.
62. Marker, S.; Le Mouël, A.; Meyer, E.; Simon, M. Distinct RNA-dependent RNA polymerases are required for RNAi triggered by double-stranded RNA versus truncated transgenes in Paramecium tetraurelia. *Nucleic acids research* **2010**, *38*, 4092–4107.
63. Bouhouche, K.; Gout, J.F.; Kapusta, A.; Bétermier, M.; Meyer, E. Functional specialization of Piwi proteins in Paramecium tetraurelia from post-transcriptional gene silencing to genome remodelling. *Nucleic acids research* **2011**, *39*, 4249–4264.
64. Martin, M. Cutadapt removes adapter sequences from high-throughput sequencing reads. *2011* **2011**, *17*.

Bibliography

33. Holoch, D.; Moazed, D. RNA-mediated epigenetic regulation of gene expression. *Nature Reviews Genetics* **2015**, *16*, 71–84.
34. Neeb, Z.T.; Nowacki, M. RNA-mediated transgenerational inheritance in ciliates and plants. *Chromosoma* **2017**, pp. 1–9.
35. Sarkies, P.; Miska, E.A. Small RNAs break out: the molecular cell biology of mobile small RNAs. *Nature Reviews Molecular Cell Biology* **2014**, *15*, 525–535.
36. Bühler, M.; Verdel, A.; Moazed, D. Tethering RITS to a nascent transcript initiates RNAi-and heterochromatin-dependent gene silencing. *Cell* **2006**, *125*, 873–886.
37. Simmer, F.; Buscaino, A.; Kos-Braun, I.C.; Kagansky, A.; Boukaba, A.; Urano, T.; Kerr, A.R.; Allshire, R.C. Hairpin RNA induces secondary small interfering RNA synthesis and silencing in trans in fission yeast. *EMBO reports* **2010**, *11*, 112–118.
38. Hollick, J.B. Paramutation and related phenomena in diverse species. *Nature reviews Genetics* **2017**, *18*, 5–23.
39. Sapetschnig, A.; Sarkies, P.; Lehrbach, N.J.; Miska, E.A. Tertiary siRNAs mediate paramutation in C. elegans. *PLoS genetics* **2015**, *11*, e1005078.
40. De Vanssay, A.; Bougé, A.L.; Boivin, A.; Hermant, C.; Teysset, L.; Delmarre, V.; Antoniewski, C.; Ronsseray, S. Paramutation in Drosophila linked to emergence of a piRNA-producing locus. *Nature* **2012**, *490*, 112–115.
41. Tricker, P.J. Transgenerational inheritance or resetting of stress-induced epigenetic modifications: two sides of the same coin. *Frontiers in plant science* **2015**, *6*.
42. Simon, M.; Plattner, H. Unicellular eukaryotes as models in cell and molecular biology: critical appraisal of their past and future value. *Int Rev Cell Mol Biol* **2014**, *309*, 141–198.
43. Galvani, A.; Sperling, L. RNA interference by feeding in Paramecium. *TRENDS in Genetics* **2002**, *18*, 11–12.
44. Ruiz, F.; Vayssié, L.; Klotz, C.; Sperling, L.; Madeddu, L. Homology-dependent gene silencing in Paramecium. *Molecular biology of the cell* **1998**, *9*, 931–943.
45. Garnier, O.; Serrano, V.; Duharcourt, S.; Meyer, E. RNA-mediated programming of developmental genome rearrangements in Paramecium tetraurelia. *Molecular and cellular biology* **2004**, *24*, 7370–7379.
46. Galvani, A.; Sperling, L. Transgene-mediated post-transcriptional gene silencing is inhibited by 3 non-coding sequences in Paramecium. *Nucleic acids research* **2001**, *29*, 4387–4394.
47. Marker, S.; Le Mouel, A.; Meyer, E.; Simon, M. Distinct RNA-dependent RNA polymerases are required for RNAi triggered by double-stranded RNA versus truncated transgenes in Paramecium tetraurelia. *Nucleic Acids Res* **2010**, *38*, 4092–107.

48. Marker, S.; Carradec, Q.; Tanty, V.; Arnaiz, O.; Meyer, E. A forward genetic screen reveals essential and non-essential RNAi factors in Paramecium tetraurelia. *Nucleic acids research* **2014**, *42*, 7268–7280.
49. Götz, U.; Marker, S.; Cheaib, M.; Andresen, K.; Shrestha, S.; Durai, D.A.; Nordström, K.J.; Schulz, M.H.; Simon, M. Two sets of RNAi components are required for heterochromatin formation in trans triggered by truncated transgenes. *Nucleic acids research* **2016**, *44*, 5908–5923.
50. Beale, G.H.; others. The genetics of Paramecium aurelia. *The genetics of Paramecium aurelia.* **1954**.
51. Cheaib, M.; Dehghani Amirabad, A.; Nordstrom, K.J.; Schulz, M.H.; Simon, M. Epigenetic regulation of serotype expression antagonizes transcriptome dynamics in Paramecium tetraurelia. *DNA Res* **2015**, *22*, 293–305.
52. Meyer, E.; Chalker, D.L.; others. Epigenetics of ciliates. *Epigenetics* **2007**, pp. 127–150.
53. Lepere, G.; Nowacki, M.; Serrano, V.; Gout, J.F.; Guglielmi, G.; Duharcourt, S.; Meyer, E. Silencing-associated and meiosis-specific small RNA pathways in Paramecium tetraurelia. *Nucleic acids research* **2008**, *37*, 903–915.
54. Ni, J.Z.; Kalinava, N.; Chen, E.; Huang, A.; Trinh, T.; Gu, S.G. A transgenerational role of the germline nuclear RNAi pathway in repressing heat stress-induced transcriptional activation in C. elegans. *Epigenetics & chromatin* **2016**, *9*, 3.
55. Oberti, D.; Biasini, A.; Kirschmann, M.A.; Genoud, C.; Stunnenberg, R.; Shimada, Y.; Bühler, M. Dicer and HSP104 function in a negative feedback loop to confer robustness to environmental stress. *Cell reports* **2015**, *10*, 47–61.
56. Cheaib, M.; Dehghani Amirabad, A.; Nordström, K.J.; Schulz, M.H.; Simon, M. Epigenetic regulation of serotype expression antagonizes transcriptome dynamics in Paramecium tetraurelia. *DNA Research* **2015**, *22*, 293–305.
57. Szittya, G.; Silhavy, D.; Molnár, A.; Havelda, Z.; Lovas, Á.; Lakatos, L.; Bánfalvi, Z.; Burgyán, J. Low temperature inhibits RNA silencing-mediated defence by the control of siRNA generation. *The EMBO Journal* **2003**, *22*, 633–640.
58. Voinnet, O. RNA silencing as a plant immune system against viruses. *TRENDS in Genetics* **2001**, *17*, 449–459.
59. Dalmay, T.; Hamilton, A.; Rudd, S.; Angell, S.; Baulcombe, D.C. An RNA-dependent RNA polymerase gene in Arabidopsis is required for posttranscriptional gene silencing mediated by a transgene but not by a virus. *Cell* **2000**, *101*, 543–553.
60. Romon, M.; Soustre-Gacougnolle, I.; Schmitt, C.; Perrin, M.; Burdloff, Y.; Chevalier, E.; Mutterer, J.; Himber, C.; Zervudacki, J.; Montavon, T.; others. RNA silencing is resistant to low-temperature in grapevine. *PloS one* **2013**, *8*, e82652.
61. Sandoval, P.Y.; Swart, E.C.; Arambasic, M.; Nowacki, M. Functional diversification of Dicer-like proteins and small RNAs required for genome sculpting. *Developmental cell* **2014**, *28*, 174–188.
62. Marker, S.; Le Mouël, A.; Meyer, E.; Simon, M. Distinct RNA-dependent RNA polymerases are required for RNAi triggered by double-stranded RNA versus truncated transgenes in Paramecium tetraurelia. *Nucleic acids research* **2010**, *38*, 4092–4107.
63. Bouhouche, K.; Gout, J.F.; Kapusta, A.; Bétermier, M.; Meyer, E. Functional specialization of Piwi proteins in Paramecium tetraurelia from post-transcriptional gene silencing to genome remodelling. *Nucleic acids research* **2011**, *39*, 4249–4264.
64. Martin, M. Cutadapt removes adapter sequences from high-throughput sequencing reads. *2011* **2011**, *17*.

© 2018 by the authors. Submitted to *Genes* for possible open access publication under the terms and conditions of the Creative Commons Attribution license (http://creativecommons.org/licenses/by/4.0/)
