# Supplementary figures and images for: Environmental Temperature Controls Accumulation of Transacting siRNAs Involved in Heterochromatin Formation
Source: Genes (Basel). 2018 Feb 21;9(2):117. doi: 10.3390/genes9020117 (PMC5852613; doi:10.3390/genes9020117)

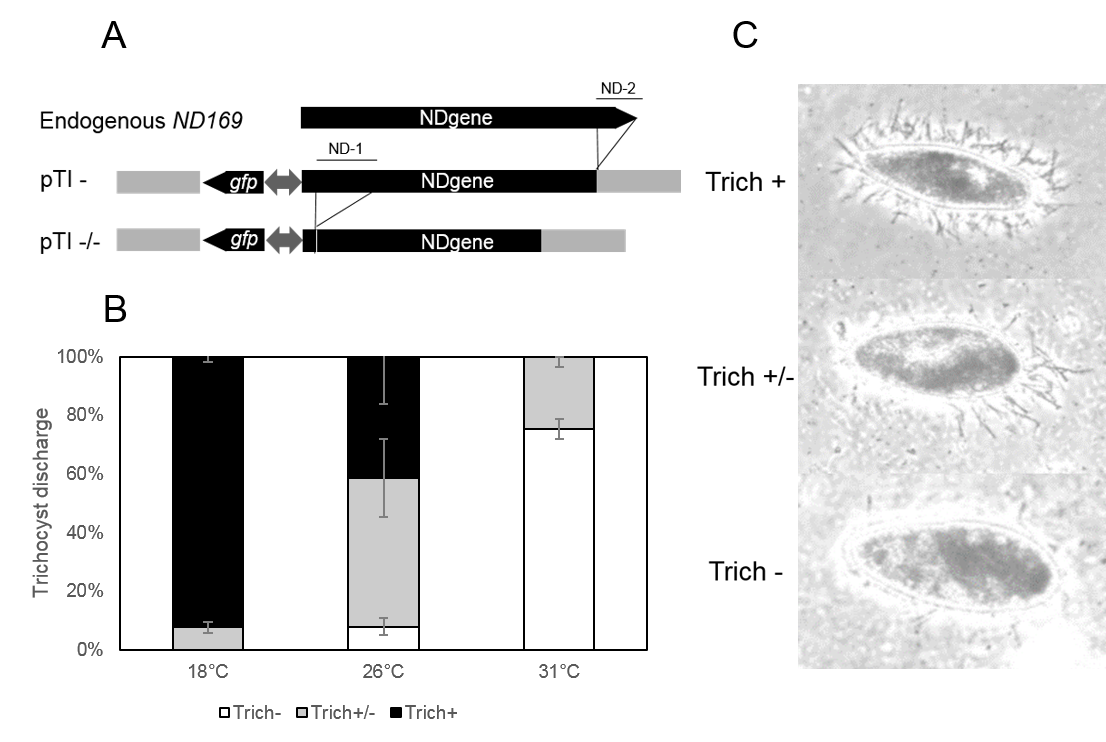

Supplement: Supplementary file 1 [file genes-09-00117-s001.zip › genes-264579/Fig_1.png]

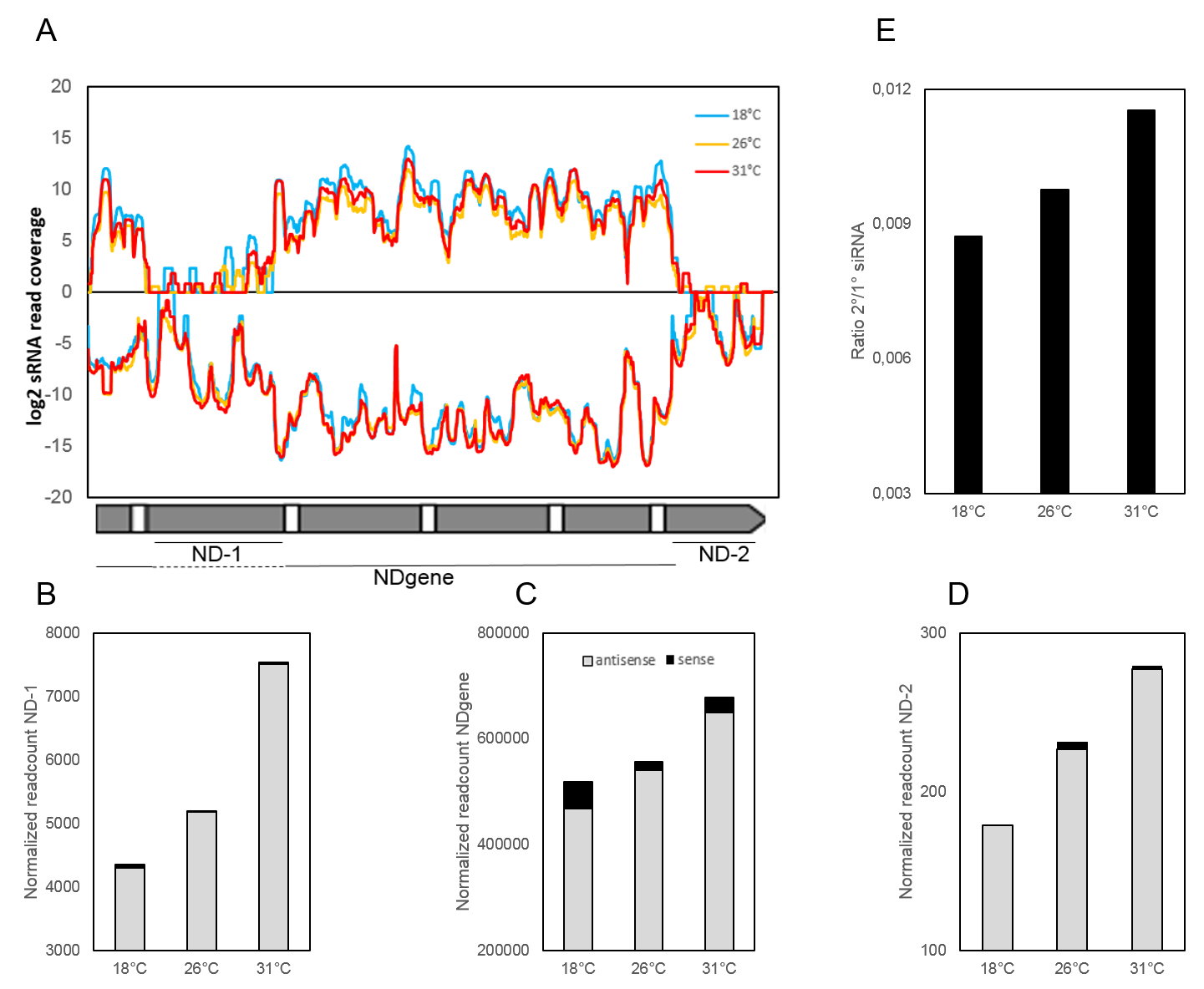

Supplement: Supplementary file 1 [file genes-09-00117-s001.zip › genes-264579/Fig_3.png]

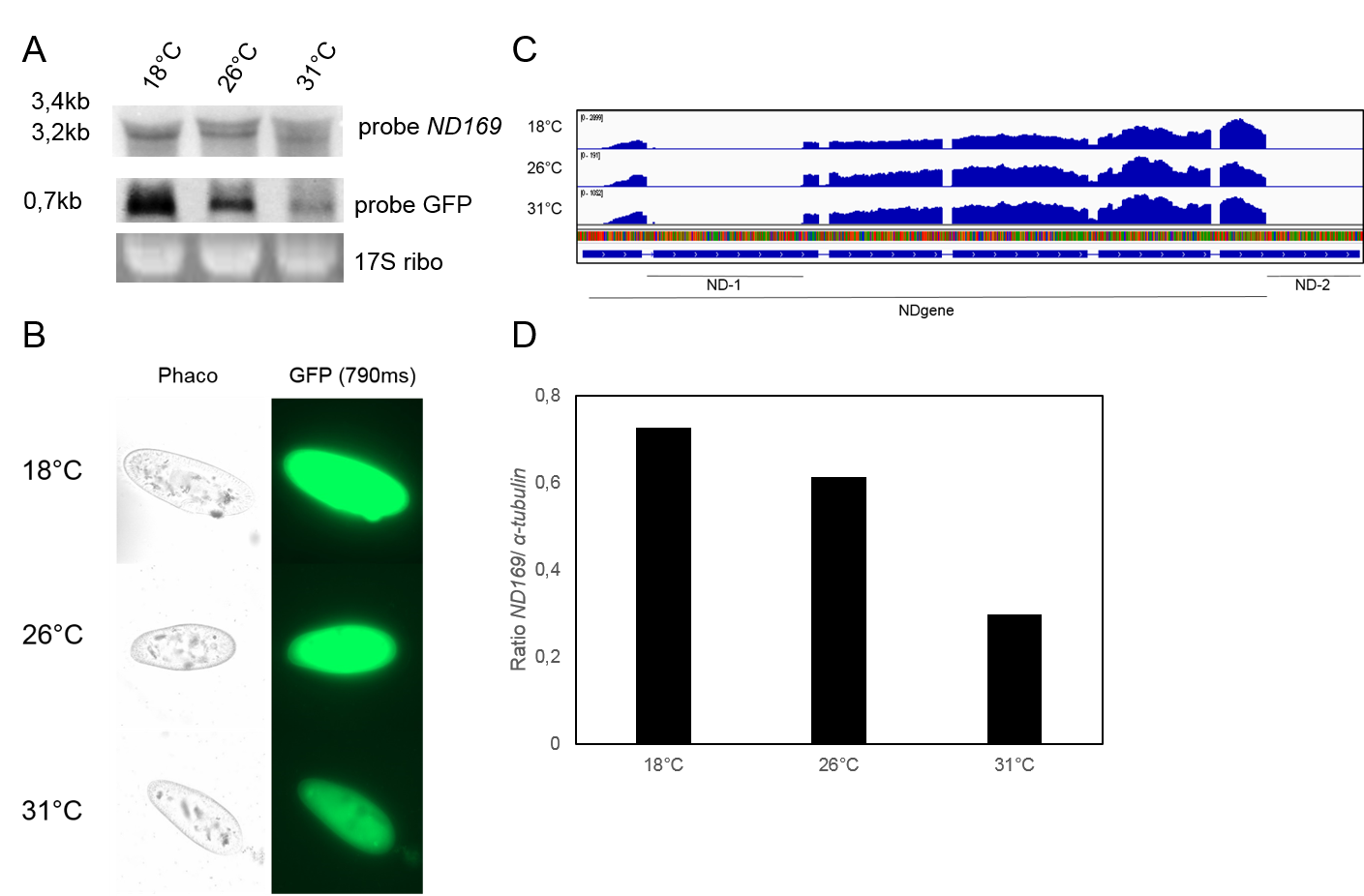

Supplement: Supplementary file 1 [file genes-09-00117-s001.zip › genes-264579/Fig_4.png]

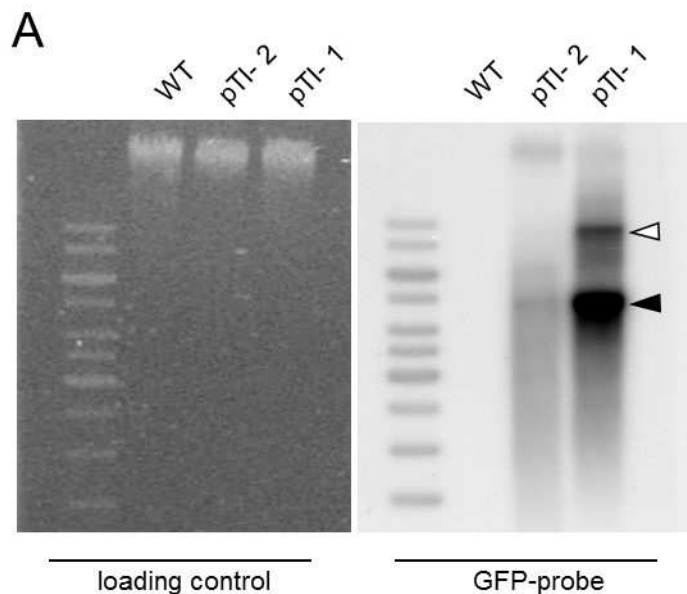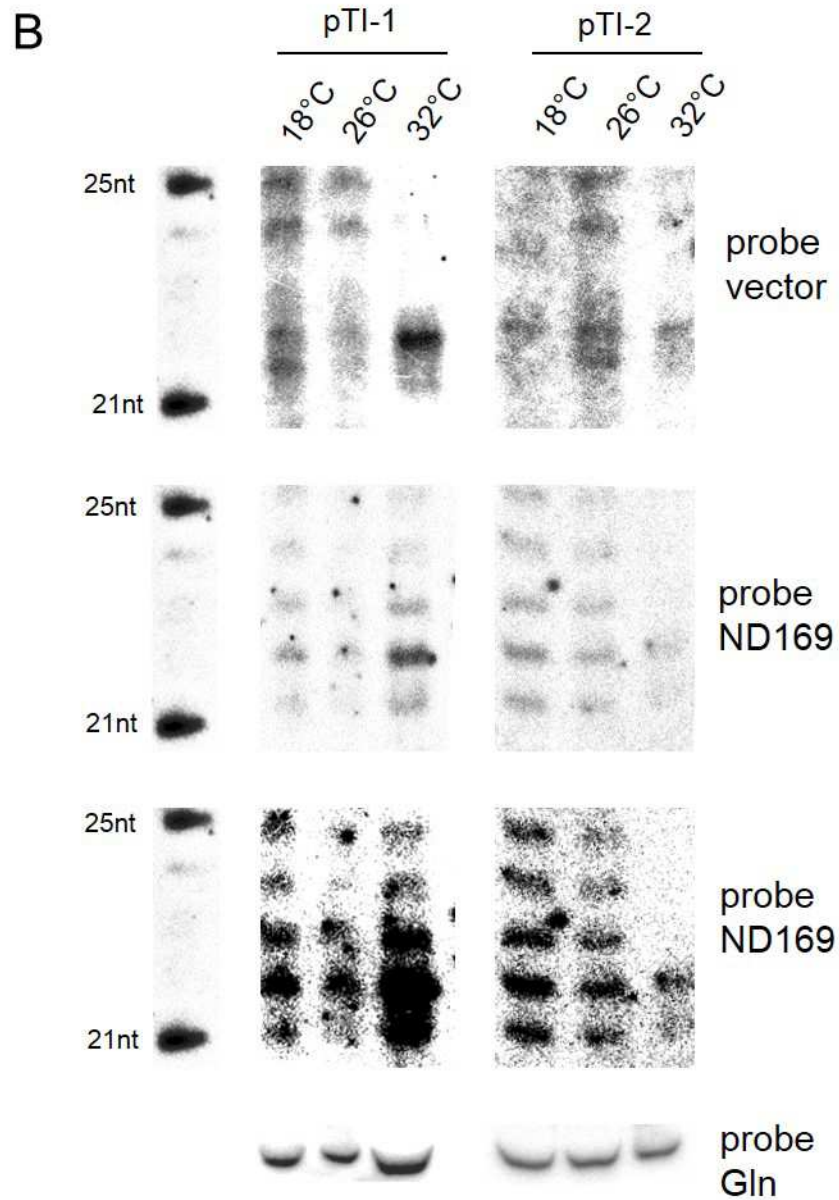

Supplement: Supplementary file 1 [file genes-09-00117-s001.zip › genes-264579/Fig2-eps-converted-to.pdf]

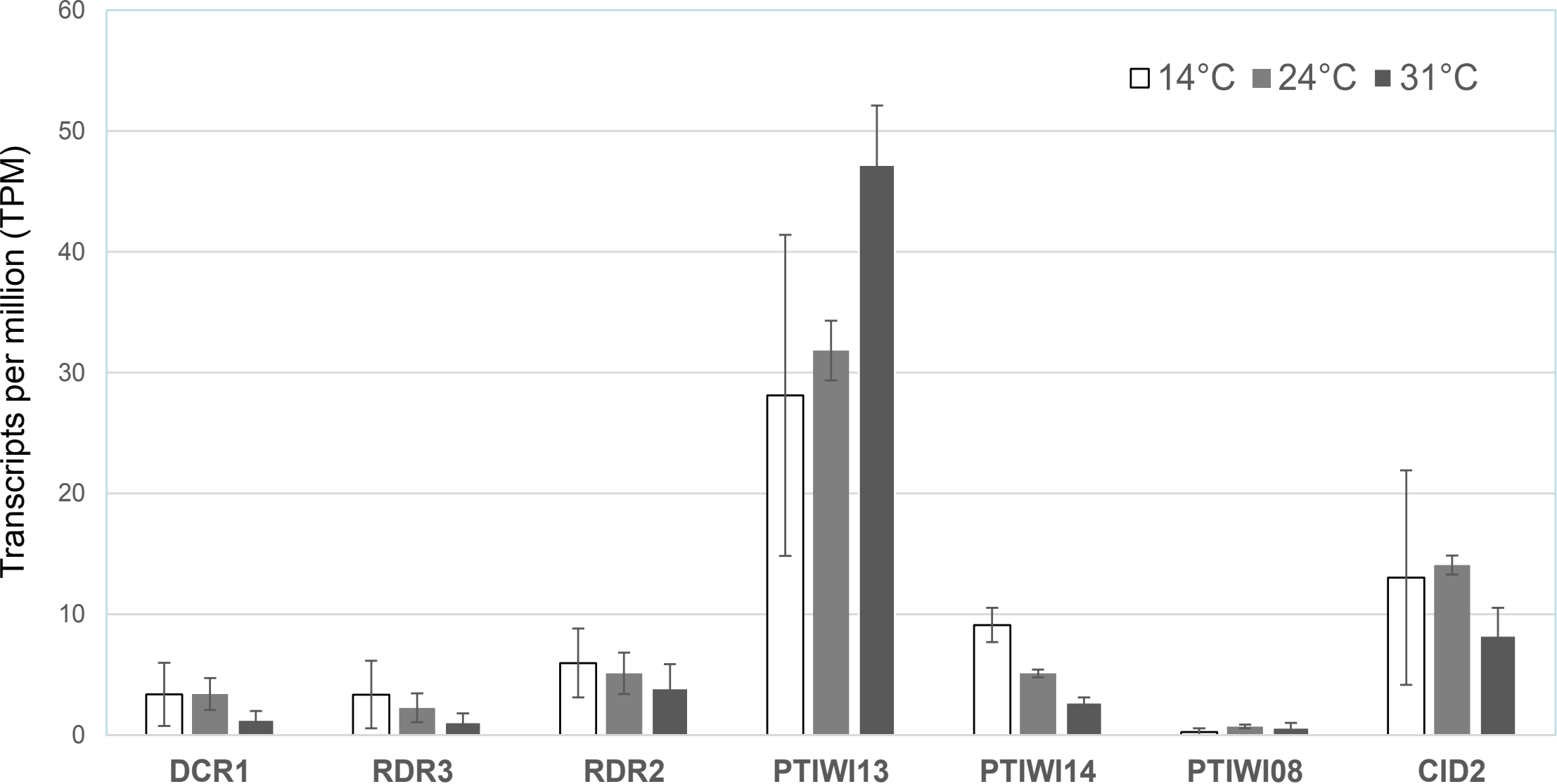

Supplement: Supplementary file 1 [file genes-09-00117-s001.zip › genes-264579/fig5-eps-converted-to.pdf]

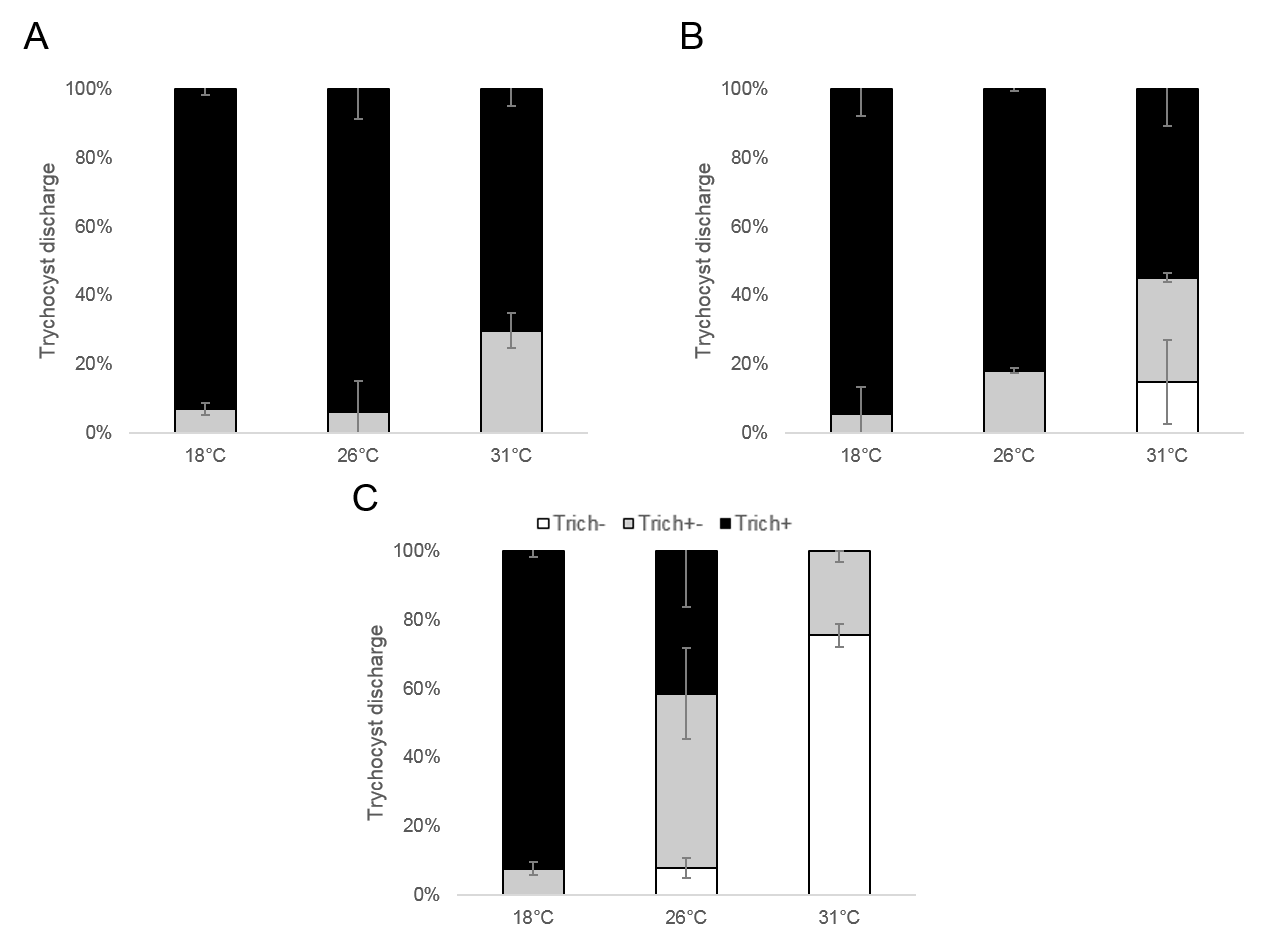

Supplement: Supplementary file 1 [file genes-09-00117-s001.zip › genes-264579/Suppl__Fig__1.png]

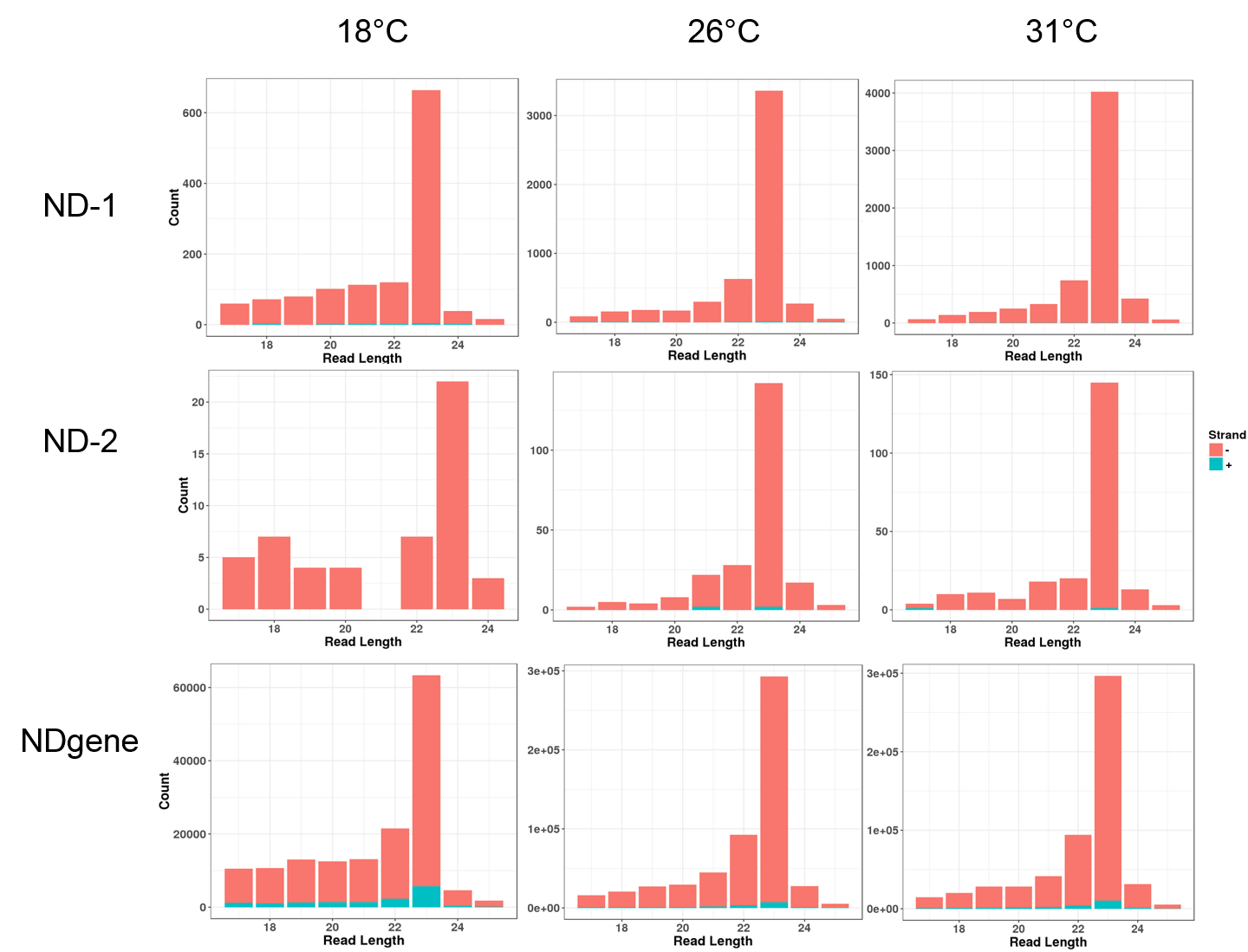

Supplement: Supplementary file 1 [file genes-09-00117-s001.zip › genes-264579/Suppl__Fig__2.png]
